# phyBWT2: phylogeny reconstruction via eBWT positional clustering

**DOI:** 10.1186/s13015-023-00232-4

**Published:** 2023-08-03

**Authors:** Veronica Guerrini, Alessio Conte, Roberto Grossi, Gianni Liti, Giovanna Rosone, Lorenzo Tattini

**Affiliations:** 1 Dipartimento di Informatica, University of Pisa, Pisa, Italy; 2 CNRS UMR 7284, INSERM U1081 Université Côte d’Azu, Nice, France

**Keywords:** Phylogeny, Partition tree, BWT, Positional cluster, Alignment-free, Reference-free, Assembly-free

## Abstract

**Background:**

Molecular phylogenetics studies the evolutionary relationships among the individuals of a population through their biological sequences. It may provide insights about the origin and the evolution of viral diseases, or highlight complex evolutionary trajectories. A key task is inferring phylogenetic trees from any type of sequencing data, including raw short reads. Yet, several tools require pre-processed input data e.g. from complex computational pipelines based on *de novo* assembly or from mappings against a reference genome. As sequencing technologies keep becoming cheaper, this puts increasing pressure on designing methods that perform analysis directly on their outputs. From this viewpoint, there is a growing interest in alignment-, assembly-, and reference-free methods that could work on several data including raw reads data.

**Results:**

We present phyBWT2, a newly improved version of phyBWT (Guerrini et al. in 22nd International Workshop on Algorithms in Bioinformatics (WABI) 242:23–12319, 2022). Both of them directly reconstruct phylogenetic trees bypassing both the alignment against a reference genome and de novo assembly. They exploit the combinatorial properties of the extended Burrows-Wheeler Transform (eBWT) and the corresponding eBWT positional clustering framework to detect relevant blocks of the longest shared substrings of varying length (unlike the *k*-mer-based approaches that need to fix the length *k* a priori). As a result, they provide novel alignment-, assembly-, and reference-free methods that build partition trees without relying on the pairwise comparison of sequences, thus avoiding to use a distance matrix to infer phylogeny. In addition, phyBWT2 outperforms phyBWT in terms of running time, as the former reconstructs phylogenetic trees step-by-step by considering multiple partitions, instead of just one partition at a time, as previously done by the latter.

**Conclusions:**

Based on the results of the experiments on sequencing data, we conclude that our method can produce trees of quality comparable to the benchmark phylogeny by handling datasets of different types (short reads, contigs, or entire genomes). Overall, the experiments confirm the effectiveness of phyBWT2 that improves the performance of its previous version phyBWT, while preserving the accuracy of the results.

**Supplementary Information:**

The online version contains supplementary material available at 10.1186/s13015-023-00232-4.

## Background

Phylogenetics concerns the study of the evolutionary history and the relationships among individuals or groups of individuals, e.g., species or several populations of one species. These relationships are inferred from heritable traits or, for instance, DNA sequences. Phylogenies, in the form of rooted or unrooted trees, can be used for several purposes: to reconstruct the ancestry of the species (or other taxa) on the tree of life, to understand the epidemiological dynamics of pathogens, and to identify and study complex evolutionary events such as hybridisation [1, 2], introgression [3], and horizontal gene transfer [4]. Thus, they are successfully employed in almost every branch of biology, including e.g. population genomics and metagenomics, ecology, and biogeography [5]. Phylogeny has also important applications in the medical field, including for instance epidemiology, drug discovery and drug design. Concerning public health, pathogen outbreaks can be studied by molecular phylogenetic analysis. Indeed, the analysis of the epidemiological link between genetic sequences of a pathogen can be useful for understanding the possible sources of transmission.

A vast array of techniques for inferring phylogeny has been developed over the years [6]. Sequence-based methods analyze the DNA or RNA sequences of the taxa, and are based on their similarity or dissimilarity detection. Most of them rely on a distance matrix by computing the pairwise evolutionary distances between every pair of input sequences. Standard algorithms, such as the neighbour-joining algorithm [7], are then applied to the distance matrix to perform the tree reconstruction.

A key aspect is how to compute these evolutionary distances. Sequence alignment is often employed in distance computation, performed on either entire sequences or parts of them, with the optional usage of a reference genome. However, with the advent of high-throughput sequencing technologies and the completion of various genome projects, the amount of whole-genome sequencing data available has increased and a new era for phylogeny started. Owing to the rising cost of the alignment task, alignment-free approaches for quantifying the similarity/dissimilarity between sequences have been introduced. An advantage of these approaches is that they are robust for recombination and shuffling events [8–10]. As the majority of alignment-free approaches for phylogenetic reconstruction performs a preliminary extraction of the *k*-mers (i.e. substrings of length *k*) from the input sequences, they can analyze directly the reads obtained from the sequencing platforms, thus avoiding the assembly of these reads and the extraction of the *k*-mers from the assembly.

*Our contribution* In this paper we present phyBWT2, a new version of our tool called phyBWT and previously introduced in [11] to reconstruct a phylogenetic tree for a set of taxa. The worst-case running time of phyBWT2 is $O(N \ell )$ for $\ell$ taxa of total length *N*, using $O(N + \ell ^2)$ space. Like its predecessor, phyBWT2 combines many features in a single new method to reconstruct a phylogenetic tree starting from any type of data, e.g. assembled sequences as well as raw reads. Firstly, phyBWT2 is alignment-, assembly-, and reference-free, and thus it can work directly on raw sequencing reads. Secondly, it does not need a distance matrix as it does not rely on the pairwise comparison of sequences. Moreover, it exploits the combinatorial properties of the *positional clustering* framework recently introduced in [12], overcoming the limitations of employing *k*-mers with fixed size *k* a priori.

The contribution of our approach is twofold, theoretical as well as practical. To the best of our knowledge, both phyBWT and phyBWT2 are the first to apply the properties of the Extended Burrows-Wheeler Transform (eBWT), employed in the positional clustering, to the idea of decomposition for phylogenetic inference. Not only they are oblivious to extra information, such as reference sequences or read mappings, but they also avoid the workload of assembling or aligning input sequences. Finally, they infer the tree structure by comparing all the sequences simultaneously and efficiently, instead of performing their pairwise comparisons: they do not reconstruct the tree in top-down or bottom-up directions, rather they refine the current structure simultaneously in both directions (so bottom up and top down are special cases of this more general reconstruction).

Despite these common ideas behind them, phyBWT2 improves over phyBWT in several aspects. To see why, let us briefly recall how phyBWT works. It builds the phylogenetic tree through a series of partitions performed on groups of nodes. Each partition isolates groups of taxa from the others, and phyBWT always proceeds in two opposite directions while reconstructing the tree: it goes towards the leaves by dividing each part, and towards the root by grouping the parts. Each part actually generates a node of the phylogeny tree.

In phyBWT2, the tree reconstruction strategy is different, as phyBWT2 does not consider a single partition at time, but it simultaneously handles several compatible cuts that correspond to an unrefined tree. The general idea is that an unrefined partition tree can be refined by taking one of its nodes and restricting phyBWT2 ’s attention to just the groups of taxa corresponding to its children. This restriction allows phyBWT2 to look into them to estimate phylogenetic signals.

In Sect. "Experimental evaluation", we show that phyBWT2 produces phylogenic trees of quality comparable to the standard benchmarks by handling datasets of different types (short reads, contigs, or entire genomes). Furthermore, we experimentally provide evidence that phyBWT2 is more efficient than phyBWT in terms of the number of iterations performed to reconstruct a phylogenetic tree. Remarkably, phyBWT2 can keep the required data structures in external memory, thus alleviating the main memory usage. For these reasons, phyBWT2 can be considered as a replacement of our original tool phyBWT.

*State of the art* The Burrows-Wheeler Transform (BWT) [13] of a string (and the eBWT of a set of strings [14, 15]) is a suitable permutation of the symbols of the string(s), whose output shows a local similarity, i.e. symbols preceding similar contexts tend to occur in clusters. Both transformations have been intensively studied with important and successful applications in several areas. For instance, the eBWT has been used for defining alignment-free methods based on a pairwise distance matrix [14, 16–18] in order to build up a phylogenetic tree for mitochondrial DNA genomes. The positional clustering detects “interesting” blocks in the output of the eBWT  [14, 15], so that the requirement on the fixed size *k* in *k*-mers is relaxed and becomes of variable-order, not fixed a priori, in an adaptive way according to the contexts. This framework has already been used in other bioinformatics tasks, such as for detecting SNPs and INDELs in short-read datasets [19] and for lossy compression of FASTQ datasets [20].

Both phyBWT2 and phyBWT exploit the underlying properties of the eBWT: (i) the clustering effect, i.e. the fact that the eBWT tends to group together equal symbols in the transformed string that occur in similar contexts in the input string collection; (ii) the fact that if a substring *x* occurs in one or more strings, then the suffixes of the input dataset starting with *x*-occurrence are close in the sorted list of suffixes. In other words, the greater the number of these substrings shared by two taxa is, the more they are similar.

Although phyBWT2 and phyBWT do not use a distance matrix, they have some resemblance with split decomposition methods when reconstructing the tree from the information gathered through the eBWT. We recall that split decomposition relies on a solid mathematical ground [21, 22], and has been successfully applied to phylogeny [23]. The idea is to score the possible splits (i.e. bipartitions) of the taxa, and assign an isolation index to each split based on the distances in the given matrix. Compatible splits are those with an empty intersection on one of the parts in the splits, and the isolation index is treated as a priority weight in making a (greedy) choice among the splits. Compatible splits induce a tree and vice versa. However, a residual error is generated on real-world data, and a notion of weak split compatibility is preferred to create a weighted phylogeny network instead of a phylogeny tree: the shortest weighted part between any two nodes in this network gives the isolation index in the corresponding split. For $\ell$ taxa, only $O(\ell ^2)$ splits are needed for split decomposition instead of $2^\ell$ ones [21].

As the original algorithm in the seminal papers on split decomposition [21, 23] requires $O(\ell ^6)$ comparisons, further papers have addressed efficiency and extended these ideas. The recent alignment-free method SANS [24, 25] uses the notions of the split decomposition theory to greedily build a list of weakly compatible splits from which to infer phylogenies. In the list, each split has its own weight computed by counting *k*-mers that are stored in a colored de Bruijn graph [24] (this has been improved later by hashing [25], leaving the colored de Bruijn graph as input option). The calculated list of splits ordered by weight is then filtered according to two strategies that are described and implemented in the software tool SplitsTree [26]. In our experimental study, we compare the trees obtained by SANS and phyBWT2. It should be noted that SANS is also able to reconstruct phylogenetic networks whereas phyBWT2 reconstructs phylogenetic trees only.

As previously mentioned, a plethora of methods have been designed for phylogeny reconstruction (e.g. DBLP reports over 500 papers having “phylogeny” in the title). We refer the reader to [6, 27, 28] for a complete and detailed review of various methods for phylogeny estimation. We briefly mention here that among the alignment-based approaches are character-based methods [5], that generally produce alignments of the input sequences and compare all sequences simultaneously considering one character per time (e.g. using maximum parsimony or maximum likelihood).

A preliminary version of this paper appeared in [11] with limited experiments performed using our prototype tool phyBWT  .^1^ The new version phyBWT2 replaces phyBWT.

## Preliminaries

In this section, we define the general terminology we will use throughout this paper.

Let *s* be a string (also called sequence) of length *n* on the alphabet $\Sigma$. We denote the *i*-th symbol of *s* by *s*[*i*]. A *substring* of any *s* is denoted as $s[i,j] = s[i] \cdots s[j]$, with *s*[1, *j*] being called a *prefix* and $s[i,n+1]$ a *suffix* of *s*. A *k-mer* is a string of length *k*.

Let $\mathcal {S} =\{s_1,s_2,\ldots ,s_{\ell }\}$ be a collection of $\ell$ strings. We assume that each string $s_i \in \mathcal {S}$ has length $n_i$ and is followed by a special end-marker symbol $S_i[n_i+1] = \$_i$, which is lexicographically smaller than any other symbol in $\mathcal {S}$, and does not appear in $\mathcal {S}$ elsewhere.^2^

### Basic data structures

The *Burrows-Wheeler Transform* (BWT) [13] is a well-known widely used reversible string transformation that can be extended to a collection of strings. Such an extension, introduced in [14], is a reversible transformation whose output string (denoted by $\textsf {ebwt} (\mathcal {S})$) is a permutation of the symbols of all strings in $\mathcal {S}$. In [15], the authors introduced a variant of this transformation for string collection in which a distinct end-marker is appended to each string, making the collection ordered. Such transformations are known as eBWT or multi-string BWT.

The length of $\textsf {ebwt} (\mathcal {S})$ is denoted by $N=\sum _{i=1}^{\ell }(n_i+1)$, and $\textsf {ebwt} (\mathcal {S})[i]=x$, with $1\!\le \!i\!\le \!N$, if *x* circularly precedes the *i*-th suffix $S_j[k,n_j+1]$ (for some $1\le j\le \ell$ and $1\le k\le n_j\!+\!1$), according to the lexicographic sorting of the suffixes of all strings in $\mathcal {S}$.

Usually the output string $\textsf {ebwt} (\mathcal {S})$ is enhanced with the *document array* (DA) and *longest common prefix* (LCP) array of $\mathcal {S}$.

The *document array* of $\mathcal {S}$ (denoted by $\textsf {da} (\mathcal {S})$) is the array of length *N* such that $\textsf {da} (\mathcal {S})[i]=j$, with $1\le j\le \ell$ and $1 \le i \le N$, where $\textsf {ebwt} (\mathcal {S})[i]$ is a symbol of the string $s_j$.

The *longest common prefix* (LCP) array [29] of $\mathcal {S}$ is the array $\textsf {lcp} (\mathcal {S})$ of length $N+1$, such that $\textsf {lcp} (\mathcal {S})[i]$, with $2 \le i \le N$, is the length of the longest common prefix between the suffixes associated with the positions *i* and $i-1$ in $\textsf {ebwt} (\mathcal {S})$, and $\textsf {lcp} (\mathcal {S})[1] = \textsf {lcp} (\mathcal {S})[N+1]= 0$ by default. The set $\mathcal {S}$ can be omitted when it is clear from the context.

The following is an important property of the eBWT, and thus of the related data structures DA and LCP, that will be used in our method:

#### Remark 1

The eBWT, DA and LCP data structures for a subset of $\mathcal {S}$ can be obtained by linearly scanning those built for $\mathcal {S}$.

In [15], the authors prove that given a collection $\mathcal {S} =\{S_1,S_2,\ldots ,S_{\ell }\}$ of strings and $\textsf {ebwt} (\mathcal {S})$, one can obtain the eBWT of a subset $\mathcal {R}$ of $\mathcal {S}$ by removing all the characters not in $\mathcal {R}$, without constructing the eBWT from scratch, as the relative order of suffixes holds. One can obtain the DA for $\mathcal {R}$ analogously by scanning $\textsf {da} (\mathcal {S})$ and removing entries not in $\mathcal {R}$.

Similarly, one can obtain the LCP of a subset of $\mathcal {S}$ by using the properties of the LCP array: for any pair of indices $i<j$, the longest common prefix between the suffix associated with position *i* and the suffix associated with position *j* is given by $\min \{\textsf {lcp} [i+1],\ldots ,\textsf {lcp} [j]\}$.

Let $\mathcal {R}\subset \mathcal {S}$. We denote by $\textsf {ebwt} (\mathcal {S})|_{\mathcal {R}}$ (resp. $\textsf {da} (\mathcal {S})|_{\mathcal {R}}$, $\textsf {lcp} (\mathcal {S})|_{\mathcal {R}}$) the restriction of the data structure $\textsf {ebwt} (\mathcal {S})$ (resp. $\textsf {da} (\mathcal {S})$, $\textsf {lcp} (\mathcal {S})$) to the set of strings $\mathcal {R}$.

### LCP-interval and k-mer vs positional cluster

We denote by LCP-intervals  of LCP-value *k* maximal intervals [*i*, *j*] that satisfy $\textsf {lcp} (\mathcal {S})[r] \ge k$ for $i < r \le j$ (slightly different definition from [30]). The suffixes associated with LCP-intervals of LCP-value *k* have a common *k*-mer as prefix.

In any string collection, thus, LCP-intervals of LCP-value *k* are in a one-to-one correspondence with the set of all *k*-mers.

Note that the common prefix *w* in a LCP-interval is of length at least *k*, but it could be longer. So, to overcome the limitation of strategies based on LCP-intervals that require to fix the length *k*, the authors of [12, 19] introduced a new framework called “positional clustering”. In this framework the intervals do not depend on a value *k* fixed a-priori, but they are enclosed between two “local minima” in the LCP-array (thus, their boundaries are data-driven).

Crucially, the length *k* of the common prefix *w* of the suffixes inside such intervals is not the same, but it differs interval by interval. Hence, there is no one-to-one correspondence between such intervals and the set of *k*-mers.

However, as to exclude intervals corresponding to some short random contexts *w*, one needs to set a minimum length for *w*, which we denote by $k_m$.

According to [19], an *eBWT positional cluster* eBWTclust[*i*, *j*] is a maximal substring $\textsf {ebwt} [i,j]$ where $\textsf {lcp} [r] \ge k_m$, for all $i< r \le j$, and none of the indices *r*, $i < r \le j$, is a *local minimum* of the LCP array.

By definition, we have that:

#### Remark 2

Any two different eBWT positional clusters, eBWTclust[*i*, *j*] and eBWTclust $[i',j']$, such that $i\ne i'$ are disjoint, i.e. it holds that either $j<i'$ or $j'<i$.

Here, we define a local minimum of the LCP array (of length *N*) any index *i*, $1<i<N$ such that $\textsf {lcp} [i-1]>\textsf {lcp} [i]$ and $\textsf {lcp} [i]<\textsf {lcp} [i+j]$, where $j>1$ is the number of adjacent occurrences of the value $\textsf {lcp} [i]$ from position *i*. For instance, let $\textsf {lcp} =[2,1,3,3,5,4,2,2,7]$. The local minima are indices 2 and 7, corresponding to LCP values of 1 and 2, respectively.

Note that the above definition differs from that in [19], where local minima in the LCP array (of length *N*) are detected searching for indices *r* such that $\textsf {lcp} [r-1] > \textsf {lcp} [r]\le\textsf {lcp} [r+1]$, for all $1 < r \le N$. According to such definition, local minima can be detected in any non-increasing sequence where some values are repeated. For instance, for the first occurrence of 4 in the sequence 5, 4, 4, 2 yields the definition of local minimum. Therefore, the slightly different notion of local minima we use is to maximize the length of the non-increasing sequence described in the following Remark 3.

#### Remark 3

([12, Thm 3.3]) In any eBWT positional cluster, the LCP-value form a sequence of non-decreasing values followed by a (possibly empty) sequence of non-increasing values.

From the above remark follows that the length *l* of the longest common prefix shared by the suffixes associated with a eBWT positional cluster $\textsf {ebwt} [i,j]$ is given by the minimum value in $\textsf {lcp} [i+1,j]$, which could be simply obtained by taking the minimum between the values $\textsf {lcp} [i+1]$ and $\textsf {lcp} [j]$.

In general, if we set the minimum length $k_m$ equal to *k*, the set of eBWT positional clusters forms a refinement of the set of $\textsf {ebwt} [i,j]$ with [*i*, *j*] LCP-interval of LCP-value *k*.

**Fig. 1:**
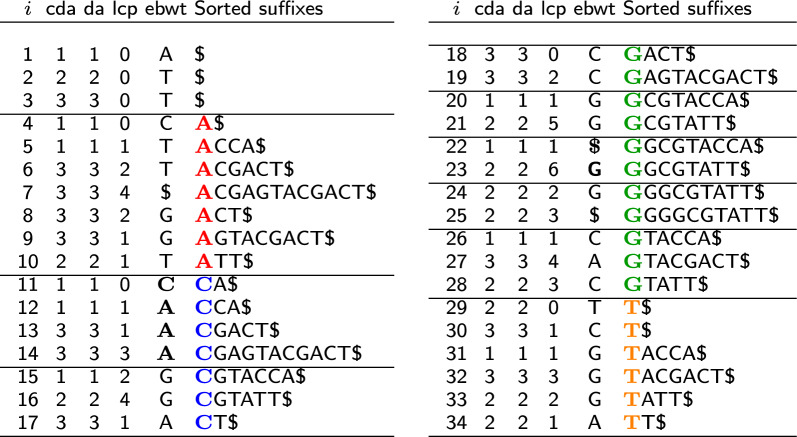
Extended Burrows-Wheeler Transform (EBWT), LCP array, and the auxiliary data structures DA and CDA for the set $\mathcal {S} =$ {GGCGTACCA, ACGAGTACGACT, GGGGCGTATT}

In fact, any $\textsf {ebwt} [i,j]$, where [*i*, *j*] is a LCP-interval, can be subdivided in correspondence of the local minima of $\textsf {lcp} [i,j]$, thus giving rise to a sequence of consecutive eBWT positional clusters (see Fig. 1). Clearly, such subdivision depends only on the trend of the LCP values inside the LCP-interval [*i*, *j*]. Hence, more than one positional cluster can be related to the same LCP-interval, and equivalently, to the same *k*-mer.

#### Example 4

(*running example*) In Fig. 1, we represent the data structures used in our tool (cda, ebwt, lcp), the auxiliary array da and the sorted list of suffixes, for the sake of clarity. The LCP-intervals of LCP-value $$k=1$$ correspond to the following intervals: [4, 10, 11, 17, 18, 28, 29, 34]. Whereas the horizontal lines delimit eBWTclust for $$ k_m = 1$$. Note that when $$k_m = k$$, the eBWTclust can refine the LCP-intervals. For example the LCP-interval [18, 28] includes five positional clusters: $$\textrm{e}{BWTclust}[18,19]$$, $$\textrm{e}{BWTclust}[20,21]$$, $$\textrm{e}{BWTclust}[22,23]$$, $$\textrm{e}{BWTclust}[24,25]$$, $$\textrm{e}{BWTclust}[26,28]$$.

## Methods

In this section, we describe the proposed method for building a phylogenetic tree where each leaf is a set of strings (sequencing reads, contigs, genome).

The idea behind our method is to reconstruct the tree through a series of refinement steps performed on groups of taxa.

The inner refinement algorithm groups together nodes whose associated strings share long common substrings of varying length which are *not* present in other nodes, and we interpret the presence of such substrings as a common feature of the group that differentiates it from the others. As mentioned in the introduction, the method is not restricted to work in a top-down or bottom-up fashion, but can act on several levels at once, according to which ones appear to be most prominent.

The final algorithm, described in Sect. "Tree reconstruction", suitably applies refinement to portions of the data, and iteratively converges to the reconstructed tree. Here refinement is a black box to group taxa, and is described in Sect. "The refinement procedure".

Given $\ell$ taxa, our method produces an unrooted tree without fixing an outgroup.

For ease of explanation, we describe the method considering the tree as rooted. Indeed, we start from a tree that has $\ell +1$ nodes: one root node, with $\ell$ children (one per taxon). Alternatively, we can imagine it as a star tree.

Before describing the reconstruction procedure, we introduce some notation and definitions.

Formally, we denote the set of leaves as $\mathcal {S} =\{S_1,S_2,\ldots ,S_{\ell }\}$ where each $S_i$ corresponds to a taxon.

The tree *T* is defined as a *partition tree* of the set $\mathcal {S}$:

- Each node of *T* corresponds to a nonempty set of taxa $S' \subseteq \mathcal {S}$
- The root of *T* corresponds to $\mathcal {S}$
- Each leaf of *T* corresponds to a distinct taxon $S_i\in \mathcal {S}$
- For each node corresponding to $S'$, its children form a partition of $S'$

It is convenient to define the operation of *adding a node* to *T* by a set: a set $S'\subseteq \mathcal {S}$ can be added to *T* only if is *compatible*, i.e., if every other node of *T* corresponds to a set $S''$ that satisfies one of these conditions: $S'' \subset S'$, $S'' \supset S'$, or $S'' \cap S' = \emptyset$ (i.e. no partial overlap between $S''$ and $S'$). If this is the case, there is only one way to add $S'$ to *T*, namely, $S'$ becomes a child of the smallest set $P \supset S'$ of *T* (by cardinality), and all the other children of *P* that are contained in $S'$ become the children of $S'$. It is easy to see that the resulting *T* is still a partition tree.

In our framework, each $S_i$ is a collection of strings, as for each taxon we can have multiple strings (like reads, contigs, a genome, and so on) possibly augmented by their reverse-complement, however in the structure of *T* it is just represented as an identifier.

Let $\mathcal {S} =\{S_1,S_2,\ldots ,S_{\ell }\}$ and each set $S_i \in \mathcal {S}$ contain $m_i$ strings, i.e. $S_i=\{s_{i,1},\ldots ,s_{i,m_i}\}$. Note that the definitions of eBWT, LCP and DA given in Sect. "Preliminaries" apply also to this case:

- $$\textsf {ebwt} (\mathcal {S})=\textsf {ebwt} (\{S_1,S_2,\ldots ,S_{\ell }\})=\textsf {ebwt} (\{s_{1,1},\ldots ,s_{1,m_1},\ldots ,s_{\ell ,1},\ldots ,s_{\ell ,m_\ell }\})$$,
- $$\textsf {lcp} (\mathcal {S})=\textsf {lcp} (\{S_1,S_2,\ldots ,S_{\ell }\})=\textsf {lcp} (\{s_{1,1},\ldots ,$$ $$s_{\ell ,m_\ell }\})$$,
- $$\textsf {da} (\mathcal {S})=\textsf {da} (\{S_1,S_2,\ldots ,S_{\ell }\})=\textsf {da} (\{s_{1,1},\ldots ,s_{\ell ,m_\ell }\})$$.

For our purposes, we extend the notion of DA to *Color Document Array* (CDA), where $\textsf {cda} (\mathcal {S})[j]=r$ if $\textsf {da} (\mathcal {S})[j]=u$ and $s_u$ belongs to the set $S_r$. In other words, we assign the same color to the strings belonging to the same set $S_r$, so we have a distinct color *r* for each set $S_r \in \mathcal {S}$.

### Example 5

(running example) In Fig. 1, cda coincides with da assuming that each taxon is a single string.

### Tree reconstruction

In this subsection we show how our method reconstructs a phylogenetic tree for $\mathcal {S}$ by suitably applying refinement. We consider refinement as a blackbox with the following properties: given a list of sets $C = C_1,\ldots , C_h$, such that any $C_k$ is a subset of $\mathcal {S}$ and disjoint from all other $C_{k'}$, for $1 \le k < k' \le h$, refinement returns a list of sets $L = L_1,\ldots , L_{|L|}$ of *compatible subsets* of $\bigcup _k C_k$: i.e., each $L_i$ is the union of some $C_k$’s, and each $L_i$ is either a subset of, a superset of, or disjoint from any other $L_j$, for $1 \le i < j \le |L|$.

The key idea is that once an intermediate partition tree is obtained, we may take one of its nodes and restrict our attention to just the groups of taxa corresponding to its children ($C_1,\ldots ,C_h$) and repeat refinement: this allows us to look at the subtree with a greater detail, by restricting the input data structures and changing the ebwt, thus bringing new tree refinements to light. This is repeated until all internal nodes in the partition tree have only two children, or no more refinements can be identified by refinement.

**Fig. 2:**
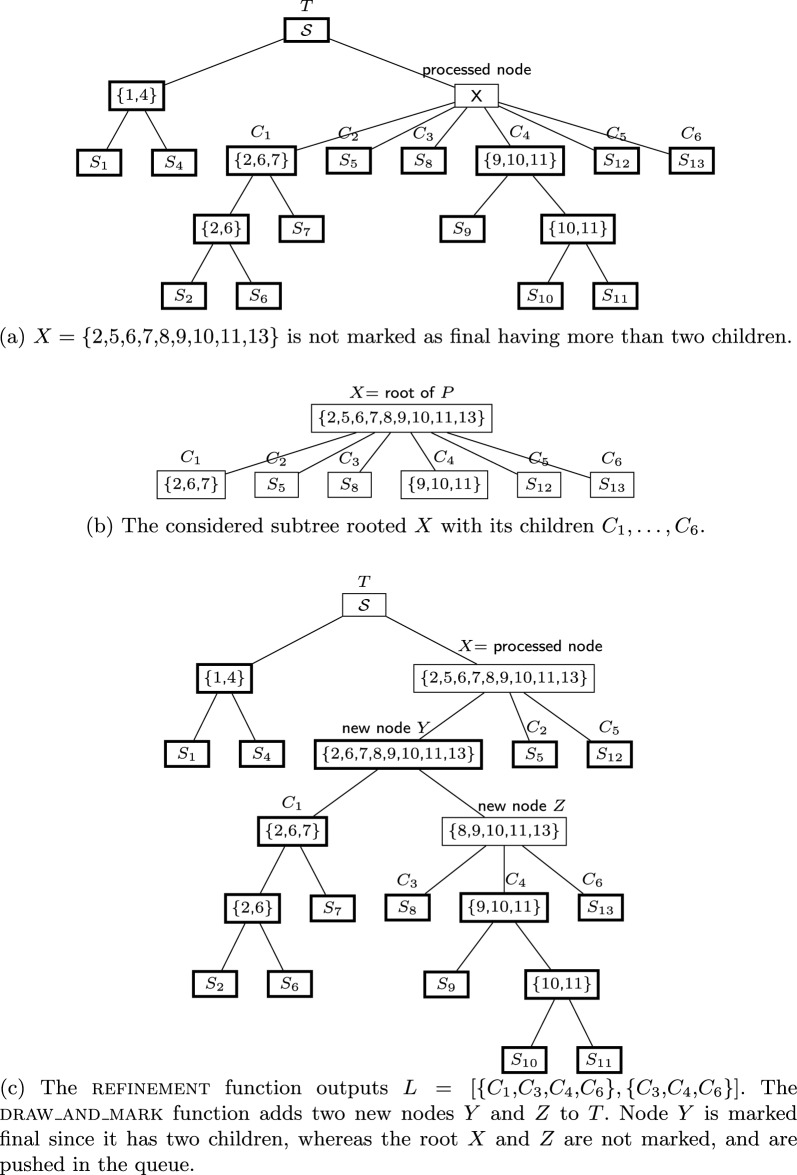
A possible iteration of Algorithm 1 where final nodes are in bold

Our algorithm is described in Algorithm 1, and one possible iteration is depicted in Fig. 2.

At the beginning we initialize the unrefined partition tree *T* (Line 1) as a rooted star with root $\mathcal {S}$ (non-final), and leaves labelled by $S_1,\ldots ,S_\ell$ marked as final. As the names suggests, *final* indicates that no more refinement is needed at that node.

The algorithm iteratively processes a non-final node *X* of *T* (Line 3), meaning that its children $C_1,\ldots ,C_h$ (which correspond to disjoint sets) are fed to refinement to create new nodes that further partition the children. All nodes produced with two children are marked final; also, if refinement fails to create new nodes, then *X* is marked final.

Line 6 calls the refinement function to create a list *L* of compatible subsets of $X = \bigcup _k C_k$, and then the draw_and_mark function is called to add the corresponding new nodes to *T* (Line 7).

By the aforementioned assumptions on the sets in *L*, they are always compatible with *T*: each $L_i$ is a subset of *X* (and all its ancestors in *T*), it is a superset of some $C_i$ (and all their descendants in *T*), and it is disjoint from all other nodes in *T*. Thus, draw_and_mark only needs to consider two cases:

- Case (*i*) *T* is not changed (i.e. the list *L* is empty): node *X* is marked as final (Line 10).
- Case (*ii*) *L* is not empty: a new internal node is created for each $L_i\in L$, by adding the set $L_i$ to the partition tree *T* as described before. A node is marked final if it has only two children; otherwise, it needs to be further refined and is added to the queue (this also applies to the node *X*).

**Fig. 3:**
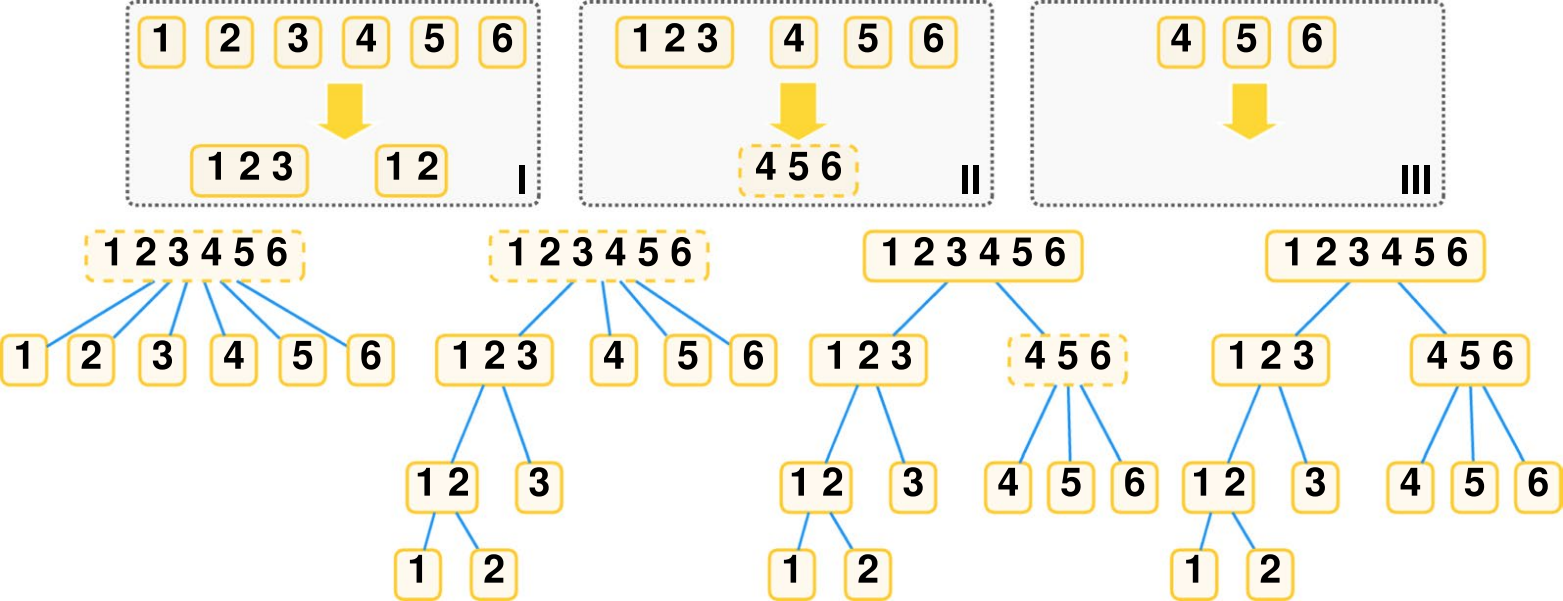
A possible execution of Algorithm 1 on a set of taxa $\mathcal {S} = \{1,2,3,4,5,6\}$. Each panel in the top shows an execution of the refinement procedure, with input on top and the list of subsets generated in the bottom. Below, the corresponding refinement of the partition tree, where dashed nodes are non-final. Note how nodes are marked final when they are leaves, or they have 2 children, or refinement fails to further cluster their children (see 4, 5, 6 in execution *III*)

Figure 2 depicts the procedure of adding nodes to *T*, while a possible execution of the algorithm is shown in Fig. 3.
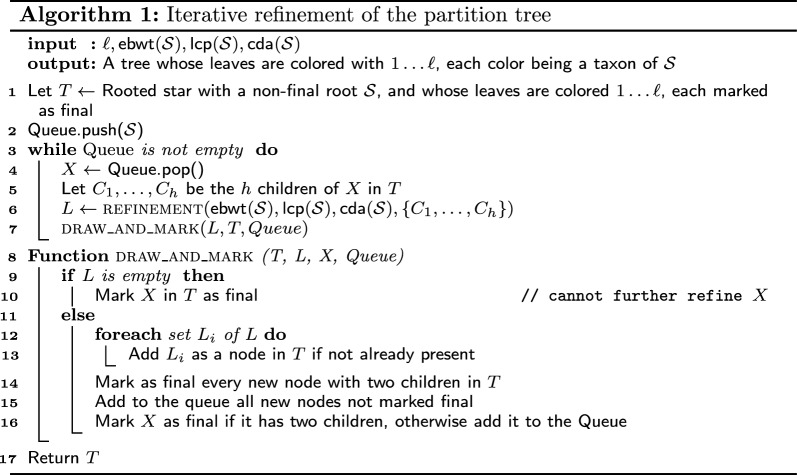

### The refinement procedure

In this subsection, we describe the approach we use as inner refinement function that starting from a set of sibling nodes $C_1,\ldots ,C_h$ returns a list *L* of compatible subsets. This is a direct evolution of the $\textsc{partition}$ procedure of phyBWT, that allows to process multiple levels at once.

According to Subsect. "Tree reconstruction", the sibling nodes $C_1,\ldots ,C_h$ of *T* to be refined correspond to some (not necessarily all) taxa: each $C_k$ can be either a leaf of *T* (thus corresponding to only one taxon) or an internal node of *T* corresponding to a subset $S'\subset \mathcal {S}$.

We can consider each $C_k$ as the set of colors (i.e. the set of taxa) to which it corresponds. Let $\mathcal {Q}=\{C_1,\ldots ,C_h\}$. We define a function $\chi _{\mathcal {Q}}$ that associates a color *r* (i.e. any taxon in $\mathcal {S}$) to the element of $\mathcal {Q}$ to which it belongs (if one exists).

#### Definition 6

Given $\mathcal {Q}=\{C_1,\ldots ,C_h\}$, we define $\chi _{\mathcal {Q}}$ from $\{1,\ldots ,\ell \}$ to $\{C_1,\ldots ,C_h\}\cup \{\emptyset \}$, such that

$$\begin{aligned} \chi _{\mathcal {Q}}(r)= \left\{ \begin{array}{ll} C_k &{} \text {if there exists}\, k\,\text { s.t.}\, r\,\text { belongs to}\, C_k\\ \emptyset &{} \text {otherwise.} \\ \end{array} \right. \end{aligned}$$

Let [*i*, *j*] be a positional cluster. Recall that we denote by eBWTclust[*i*, *j*] the concatenation of the symbols in the eBWT associated with the range [*i*, *j*] (i.e. $\textsf {ebwt} ({\mathcal {S}})[i,j]$). Then, for each eBWTclust[*i*, *j*], the corresponding interval in the CDA, $\textsf {cda} {(\mathcal {S})}[i,j]$, stores the colors (i.e. indices of the taxa) to which the symbols in eBWTclust[*i*, *j*]  belong.

#### Definition 7

An eBWTclust[*i*, *j*] is $\gamma _\mathcal {Q}$*-colored* if $\gamma _\mathcal {Q}$ is the set of elements of $\mathcal {Q}$ appearing in $\textsf {cda} {(\mathcal {S})}[i,j]$, i.e. $\gamma _{\mathcal {Q}}=\{{\chi _{\mathcal {Q}}(r)}\text {: } r\in \textsf {cda} {(\mathcal {S})}[i,j]\}$.

Note that if eBWTclust and CDA are restricted to the strings in $\mathcal {Q}$ (see Remark 1), then $\gamma _\mathcal {Q}$ contains only non-empty sets.

#### Example 8

Let $\mathcal {Q}=\{\{1,3,4\},\{2\},\{5\}, \{6\}\}$ and eBWTclust $[i,j]=ACAAGT$ with $\textsf {cda} [i,j]=[1\ 2\ 1\ 1\ 3\ 3]$. Then, eBWTclust[*i*, *j*] is $\gamma _\mathcal {Q}$-colored and $\gamma _\mathcal {Q}=\{\{1,3,4\},\{2\}\}$.

The main idea is to detect and analyze only eBWT positional clusters associated with left-maximal contexts shared by a sufficiently large number of taxa (but not by all of them).

#### Definition 9

A $\gamma _\mathcal {Q}$-colored eBWTclust[*i*, *j*] is *relevant*, if the following properties hold:

i) $\textsf {ebwt} [i,j]$ is not a concatenation of a same symbol (i.e. it is not a run),
ii) $$1<\textsf {card} (\gamma _\mathcal {Q})<h$$,
iii) $\textsf {card} (\textsf {cda} [i,j]\cap C_k)\ge \tau \cdot {\textsf {card} (C_k)}$, for all $C_k\in \gamma _\mathcal {Q}$ and some $0<\tau \le 1$.

In *ii*), we cut off the eBWT positional clusters associated with left-maximal contexts shared by only one element of $\mathcal {Q}$ or by all of them. Indeed, such contexts provide no significant information about how to group together elements of $\mathcal {Q}$.

In *iii*), we require that any element $C_k$ in $\gamma _\mathcal {Q}$ is sufficiently supported, i.e. the number of colors of $C_k$ appearing in $\textsf {cda} [i,j]$ is “large enough”. Formally, we define a *support threshold* $\tau$ ($0<\tau \le 1$) that determines the minimum required portion for each $C_k\in \gamma _\mathcal {Q}$ to be in $\textsf {cda} [i,j]$. Intuitively, the support threshold guarantees that all the elements of $\mathcal {Q}$ appearing in any eBWTclust[*i*, *j*] are sufficiently represented. In fact, when $\tau$ approaches the value of 1, all the elements of the subset $C_k$ are required to be in the cluster. In other words, we aim at measuring how similar the shared history of the phylogeny is in terms of common substrings. On the other hand, when $\tau$ approaches the value of 0, at least one of the elements of the subset $C_k$ is required to be in the cluster considered. Thus, we are observing how similar all the evolution events are. That provides two different viewpoints of their phylogenetic relationships.

#### Example 10

(Continued from Example 8) Let $\mathcal {Q}=\{\{1,3,4\},\{2\},\{5\}, \{6\}\}$ and $\tau =0.5$. The eBWTclust $[i,j]=ACAAGT$ with $\textsf {cda} [i,j]=[1\ 2\ 1\ 1\ 3\ 3]$ is a relevant cluster, since for any element of $\gamma _\mathcal {Q}$, its portion in $\textsf {cda} [i,j]$ is above $\tau$.

#### Example 11

(running example) We highlight in bold, in Fig. 1, the relevant eBWTclust, that are eBWTclust[11, 14] and eBWTclust[22, 23]. Every other eBWTclust is either a run of a same symbol or the associated $\textsf {cda}$ contains only one color or all of them.

Now, we use the notion of relevant eBWTclust to obtain a list of compatible subsets of $\mathcal {Q}=\{C_1,\ldots ,C_h\}$, having size at most $h-1$.

The whole strategy is summarized in the following three steps:

1. Scan input data structures computed on $\mathcal {\mathcal {S}}$, and detect only the relevant eBWTclust of the restricted $\text {eBWT} (\mathcal {\mathcal {S}})|_{Q}$ (denoted by eBWTclust $_{\mathcal {Q}}[i,j]$, for some $i<j$).
2. For each eBWTclust $_{\mathcal {Q}}[i,j]$, incrementally assign a score to the subset of $\mathcal {Q}$ corresponding to $\gamma _\mathcal {Q}$. Intuitively, we use the score to determine the order in which the subsets of $\mathcal {Q}$ must be processed to output the list *L*.
3. Output *L* by selecting *compatible* subsets of $\mathcal {Q}$ that record the highest scores.

By Remark 1, any relevant eBWTclust $_{\mathcal {Q}}$ detected at the first step can be inferred by a linear scan of the input data structures $\textsf {ebwt} (\mathcal {S}),\textsf {lcp} (\mathcal {S}),\textsf {cda} (\mathcal {S})$.

In general, any $\gamma _\mathcal {Q}$-colored eBWTclust $_{\mathcal {Q}}[i,j]$ may not be relevant, and thus, it does not provide a score to its corresponding subset $\gamma _\mathcal {Q}$. In our framework, each relevant eBWTclust $_{\mathcal {Q}}[i,j]$ contributes to the score of the subset $\gamma _\mathcal {Q}$ by one.

Steps 1 and 2 are in fact performed simultaneously, and only subsets of $\mathcal {Q}$ that provide at least one score are accounted for (without considering all the $2^h$ possibilities).

To build up the list *L* we greedily select the subsets of $\mathcal {Q}$ having a high score and that are compatible with each other. In particular, we first order the subsets obtained by the positional clustering by their score, and then, by scanning the sorted scores we consider subsets one-by-one. For each $\gamma _\mathcal {Q}$ we compute the subset $U = \bigcup \gamma _\mathcal {Q}$, and add it to *L* if it is compatible with what added so far (i.e., a subset of, superset of or disjoint from all elements in *L*).

#### Example 12

(Continued from Example 10) Suppose $\gamma _\mathcal {Q}=\{\{1,3,4\},\{2\}\}$ is found to be compatible with what we have added to *L* so far. Then we add the set $U = \{1,3,4\}\cup \{2\} = \{1,2,3,4\}$ to *L*.

We stop adding subsets to *L* in some cases: 

*i*) the maximum possible number of elements is reached (that is $h-2$), 

*ii*) the score decreases too much with respect to the highest score (e.g. two order of magnitude lower than the highest score) meaning that the subset associated is not trustworthy, 

*iii*) the number of consecutive unsuccessful attempts to add elements to *L* exceeds a threshold value *f* (we set this as $\min \{2h,100\}$, so that it scales with the size of the instance, with the limit of 100 to prevent excessive degeneration of quality).

### Complexity

We observe here how the pre-processing step of our method, which consists in building the eBWT, LCP and DA data structures, can be computed in time and space linear in the number *N* of all symbols of the strings in $\mathcal {S}$.

The refinement procedure described in Subsect. "The refinement procedure" can be computed in *O*(*N*) time and space: Indeed, given $\mathcal {Q}=\{C_1,...,C_h\}$ and $X=\bigcup \{C_1,...,C_h\}$, the eBWT (resp. LCP and CDA) for $X\subseteq \mathcal {S}$ can be deduced in linear time in the length *N* of $\textsf {ebwt} (\mathcal {S})$ (resp. $\textsf {lcp} (\mathcal {S})$ and $\textsf {cda} (\mathcal {S})$), including at the same time the detection of all positional clusters [12]. Given an element of a eBWTclust[*i*, *j*] and $\tau$, we can determine in *O*(1) time its color using the CDA; as we can pre-compute the size of all $C_i$, this lets us easily determine the $\gamma _\mathcal {Q}$-coloring of the cluster and whether it is relevant or not (Definitions 7 and 9) in time proportional to the cluster’s length. Overall, detecting positional clusters and assigning scores to subsets of $\mathcal {Q}$ has a total cost of *O*(*N*) time.

While potentially there could be up to $2^h \le 2^\ell$ possible subsets of $\mathcal {Q}$, we observe that each positional cluster can in fact define *at most* one of them, of size not greater than the length of the cluster. It follows that the list of subsets of $\mathcal {Q}$, from which we select the elements of *L*, has $<N$ elements, and the sum of their sizes is too at most *N*.

Next, the algorithm sorts by score the subsets of $\mathcal {Q}$ found by the positional clustering, which using a bucket sort takes *O*(*N*) time. Finally, we need to scan the sorted subsets as to obtain the output list *L* of compatible subsets, and insert new nodes in the partition tree *T* (draw_and_mark procedure).

To check whether a subset $Q'\subseteq \mathcal {Q}$ is compatible with the ones inserted in *T* so far, we proceed as follows: taken any element $y\in Q'$, take the leaf-to-root path from *y* to the root,^3^ and consider for each node the cardinality of its set. If $Q'$ is compatible, it must be inserted at one specific point in this path, i.e., where the cardinality of the lower node is $< |Q'|$ and that of the upper node is $>|Q'|$ (the path has length $\le \ell$ and we only need to scan it once to find the spot, so this can be obtained in $O(\ell )$ time). At this point, we identified the potential parent *P* of $Q'$, and we only need to verify that indeed $Q'\subset P$ (*O*(|*P*|) time), and that, for each child $P_i$ of *P*, either $P_i\subset Q'$ or $|P\cap Q'| = 0$; this latter step also takes $O(\sum |Q'|) = O(|P|)$ time, with $|P|\le \ell$ since $P\subseteq \mathcal {S}$. This means we can identify whether $Q'$ is compatible, and in case already identify the nodes that should become children of $Q'$, in $O(\ell )$ time.

Since the maximum number of subsets we analyze from the sorted list is limited by $f\cdot s$, where $s\le h$ is the number of successful insertions in *L*, and *f* the limit of consecutive failures allowed, the total cost of this step is $O(fs\ell )$, meaning that the total cost of each execution of refinement is $O(N+fs\ell )$.

The number of executions of refinement is bounded by the final number of nodes in *T*, that is $O(\ell )$. Furthermore, the total cost of the $O(fs\ell )$ factors can be amortized to $O(f\ell ^2)$ in that only up to $\ell$ successful insertions can be performed in total on *T*. It follows that the total cost is bounded by $O(N\ell + f\ell ^2)$. Finally, as *f* is a constant (no greater than 100) and $\ell \le N$ since each taxon has a positive length, we have $O(N\ell + f\ell ^2) = O(N\ell )$.

As for the space requirement, it is that of refinement plus the maximum size of the Queue and the tree *T*: refinement requires *O*(*N*) space for the ebwt structures; the Queue only holds up to $\ell$ pointers to the tree *T*, and the latter tree has $O(\ell )$ nodes each of size $O(\ell )$. The following holds:

#### Lemma 13

Given a set $\mathcal {S}$ of $\ell$ taxa, whose total length is *N*, phyBWT2 reconstructs a phylogenetic tree for $\mathcal {S}$ in $O(N\ell )$ time and $O(N + \ell ^2)$ space.

We observe that *N* is the dominant factor in this complexity, as the length of the strings representing a taxon is -in known applications- many orders of magnitude greater than the number $\ell$ of taxa.

Furthermore, $O(N\ell )$ time corresponds to the worst case in which the refinement procedure only generates one new node each time. As refinement is able to create nodes on various levels at once, this is not the expected behaviour: as showcased in Sect. "Experimental evaluation" the number of calls of the refinement procedure is far less than $\ell$ in practice.

## Experimental evaluation

In this section we test the performance of phyBWT2 for reconstructing phylogenetic trees from short-reads and de novo assembled sequences. Indeed, as for the previous version phyBWT, its usage is not limited to a particular type of input since both types of data are accepted as input. However, the diversity between the two type of input data requires a tuning of the parameters ($k_{min}$ and $\tau$).

For comparison, we selected the recently introduced tool SANS [24, 25] since it shares several features with phyBWT2: both are whole-genome based, alignment- and reference-free approaches for phylogenetic reconstruction that do not use or produce pairwise comparisons of the sequences or their characteristics. We used the latest version^4^ of SANS [25], a stand-alone re-implementation of the theoretical approach presented in [24] that improves both running time and memory usage.

Differently from phyBWT2, SANS is based on the construction of a list of splits obtained by computing all the *k*-mers of the dataset, which are either directly extracted or stored in a colored de Bruijn graph. Thus, it requires to fix a-priori the value *k*. Then, the list of splits is post-processed according to filtering strategies (options proposed in SplitsTree [26]) that allow to limit the output splits in order to show phylogenetic networks or to calculate a subset of them representing a tree. For the sake of comparison, we applied to SANS the last filtering approach for drawing trees.

In our experiments, in order to improve the sensitivity of our tool (note that SANS performs implicitly this step by adding the reverse-complement of the k-mers), we added the reverse-complement of the strings to each set of taxa.

*Implementation* Our tool has been implemented in C++. All tests were done on a DELL PowerEdge R750 machine, used in non exclusive mode. Our platform is a 24-core machine with 2 Intel(R) Xeon(R) Gold 5318Y 24C/48T CPUs at 2.10 GHz, with 629 GB. The system is Ubuntu 22.04.1 LTS.

*Input and Output* phyBWT2 takes as input ebwt ($\mathcal {S}$), lcp ($\mathcal {S}$) and cda ($\mathcal {S}$), the parameter $k_m$ that is used to remove the noise during the construction of the positional clusters and the support threshold value $\tau$ in (0, 1] used for each positional cluster coloring. Such data structures can be computed via the bash script that we provide in the phyBWT2 repository. In the current implementation, the data structures are given in uncompressed form, but phyBWT2 can be adapted to directly take as input compressed data structures [31].

Our tool outputs an unrooted tree in newick format. The trees^5^ reported in this paper are drawn by using the Interactive Tree Of Life (iTOL) tool [32].

*Datasets* To show the effectiveness of our method, we have chosen six datasets with a diverse number of taxa, composition and different length of the strings (Table 1). More in details, we used six different types of datasets: i) Illumina sequencing data (short reads) for seven *S. cerevisiae* and five *S. paradoxus* strains from the study in [33]; ii) assemblies from 12 species of the genus *Drosophila* from the FlyBase database (largely accepted phylogeny shown in [34]) (also analyzed in [24]); iii) Illumina sequencing data (short reads) for 42 *S. cerevisiae* strains selected from the studies in [35, 36] and from the public repository under accession code PRJEB50706. iv) 43 HIV-1 complete genomes used in the literature [37]; v) 20 sequences from Ebolavirus genus selected in [38, 39]. vi) 27 genomes from *E. coli* and *Shigella* from the studies in [40, 41].

*Resource usage* The new version phyBWT2 improves the performance of the former version phyBWT by reducing the internal memory usage and the running time on large datasets. Indeed, phyBWT2 does not load in main memory the data structures: it performs the clustering detection by reading portions of the input data structures computed for the whole dataset and by (possibly) reducing them to a subset of strings at the same time.

**Table 1:** Datasets

Datasets	Composition	Number of taxa	Number of sequences	Number of bp
12 yeasts	Illumina paired-end reads	12	60,000,000	9,060,000,000
Drosophila	assemblies	12	121,491	4,323,268,803
42 yeasts	Illumina paired-end reads	42	119,641,704	15,664,843,102
HIV-1	genomes	43	43	388,535
Ebolavirus	genomes	20	20	378,002
*E. coli*—Shigella	genomes	27	27	132,466,506

**Table 2:** Running times and RAM both phyBWT2 and SANS

Datasets	PhyBWT2				SANS		
	Preprocessing		Phylogeny		RAM	Wall clock	
	RAM (Kb)	Wall clock (hh:mm:ss)	RAM (Kb)	Wall clock (hh:mm:ss)			
					(Kb)	(hh:mm:ss)	
12 yeasts	27,559,652	3:19:03	6752	3:12	2,779,692	35:58	
Drosophila	92,536,548	26:11	9516	8:50	30,107,232	21:04	
42 yeasts	47,696,220	(BCR+bwt2lcp) 08:06:08	1,874,388	01:26:17	14,128,724	32:48:43	(k=32)
	4,461,196	(only BCR) 16:27:45			12,368,416	45:52:01	(k=25)
HIV-1	18,996	$< 1$s	8452	$< 1$s	9,020	$< 1$s	
Ebolavirus	18,256	$< 1$s	8460	$< 1$s	9,112	$< 1$s	
*E. coli*—Shigella	5,484,644	1:21	12,648	1:38	303,868	3:06	

We also show the the resources needed to build the data structures during the preprocessing using the tool BCR [15] and bwt2lcp [46] for short reads and gsufsort [45] for long sequences. All tools were run using one core only. The wall clock time and RAM usage are taken from the output of the /usr/bin/time command.

In Table 2 we report the running times of both phyBWT2 and SANS on the tested datasets. We separate the running time required to build the data structures from the running time of phyBWT2 for several reasons.

First, the input data structures eBWT, LCP and CDA are well-known structures in string algorithms and in bioinformatics, and efficiently building these data structures is a well-studied problem ( [15, 42–49]). Thus, analyzing the best way to compute them efficiently does not fall within the goal of this paper.

Second, the data structures do not depend on the $k_m$-value used to remove the noise during the construction of the positional clusters. Therefore, they need to be built only once for each input dataset. One can try different parameters or techniques for inferring confidence values on phylogenetic trees (based on reconstructing many trees) without having to rebuild the data structures.

The last feature does not hold for *k*-mers-based methods, such as SANS, whose data structures must be recomputed when varying the input parameter *k*.

Nevertheless, we experimentally observed that the pre-processing step (see Table 2) is computationally more expensive than the phylogeny construction.

### Experiments on 12 yeasts

This dataset comprises 12 Illumina 151-bp paired-end sequencing experiments obtained from the study in [33], and deposited in the public repository SRA (Short Reads Archive) under accession code PRJNA340312. We selected seven sequencing data from the *S. cerevisiae* strains and five from the *S. paradoxus* strains. We performed adaptor-removing and quality-based trimming using trimmomatic [50], as described in [33]. Then, for each sequencing experiment, we extracted 5 million of 151-bp paired-end reads as to form a dataset with $60\times$ coverage on average per strain and a total FASTA file size of 26 GB.

**Fig. 4:**
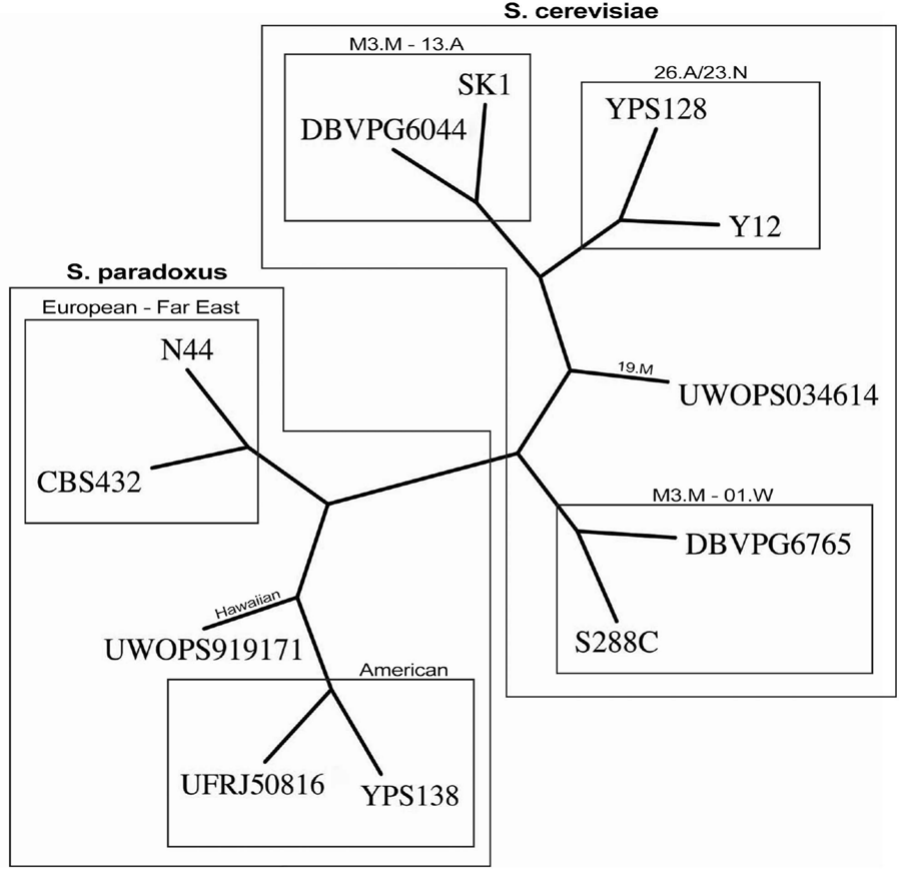
Benchmark phylogeny for the yeasts dataset. Figure redrawn from [33]

*Validation and results* As benchmark tree we used the one reported in Fig. 4 obtained from the original study [33]. Remarkably, the benchmark was built using nuclear one-to-one orthologs, i.e. the sequences of nuclear genes which are shared among (*i*) the seven *S. cerevisiae*, (*ii*) the five *S. paradoxus* strains sequenced in the study, and (*iii*) six outgroups from the *Saccharomyces* genus.

**Fig. 5:**
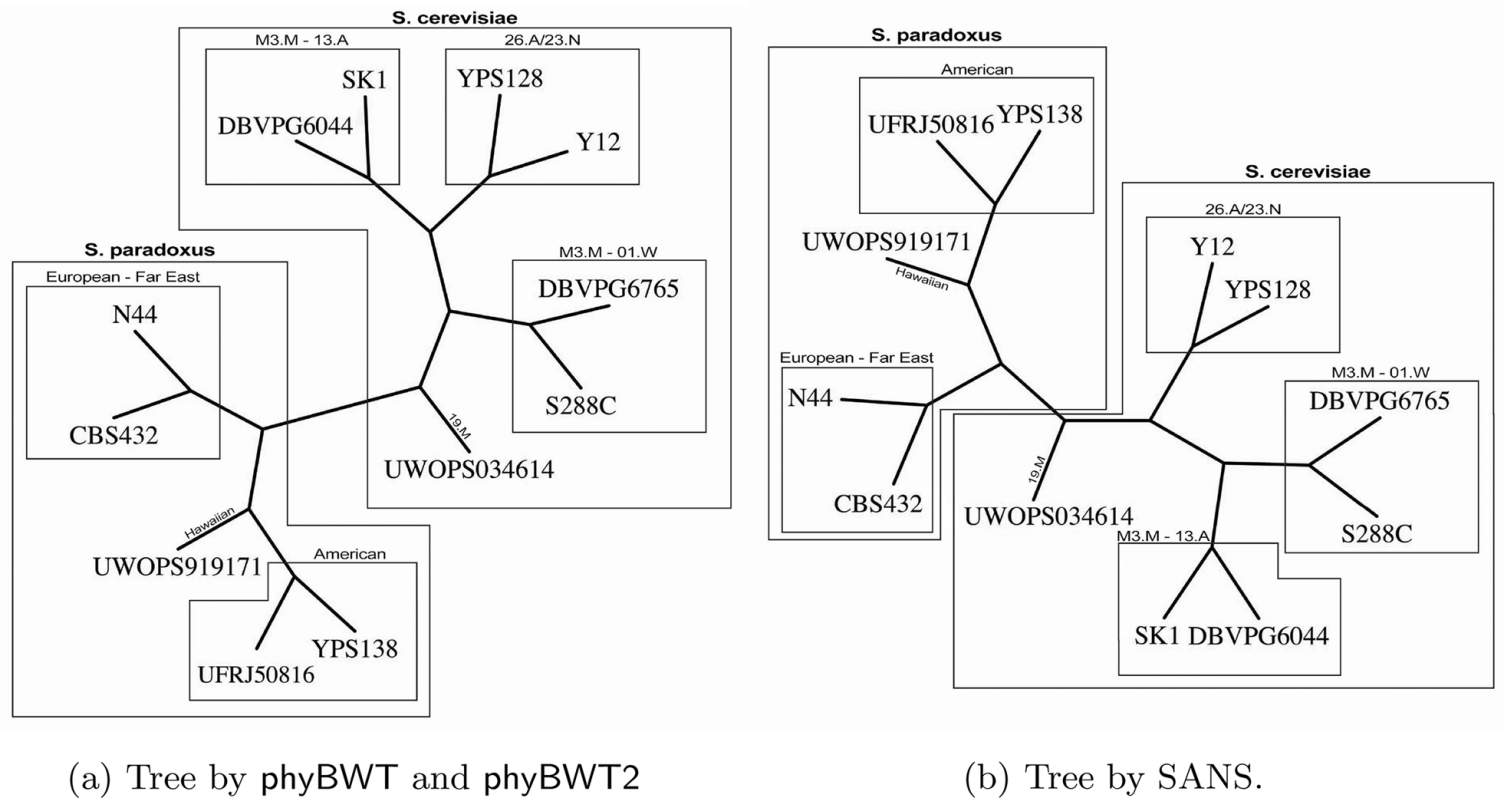
Yeasts phylogeny by phyBWT and phyBWT2 (**a**) and by SANS (**b**)

The tree depicted in Fig. 5a has been obtained by the first version phyBWT, for any $k_m\ge 14$, and $\tau =0.6$ and *t* being the number of taxa. By running phyBWT2 on the same input we obtain the same reconstructed phylogeny with similar parameters, i.e. for any $k_m>15$ and $\tau =0.6$ (parameter *t* being removed in phyBWT2). For running SANS, analogously to [11], we use default parameters that corresponds to setting the *k*-mer length to 32 (-DmaxN=32), and in addition, we set the option -f strict in order to output a tree in the Newick format (see Fig. 5b).

Both phyBWT2 and SANS separate the *S. cerevisiae* and the *S. paradoxus* strains, which show an average whole-genome sequence divergence of $\sim$ 10%. As expected, by taking into account the relatively high divergence among *S. paradoxus* strains (0.5% - 4.5%), also the same *S. paradoxus* partition is obtained. On the other hand, a few differences are shown in the *S. cerevisiae* partition which groups strains with a sequence divergence $\sim$ 0.5%. Compared to SANS, phyBWT2 produces a tree which is closer to the benchmark although the differences with the benchmark shown by both SANS and our method can be explained considering the relatively low divergence among *S. cereviasiae* strains as well as the partially admixed genomes of some of the trains (e.g. S288C and DBVPG6044) [51].

For a fair comparison, we also run SANS with different values of the input parameter *k* (i.e. by varying *k* in the range [15, 50]). The unrooted phylogenetic tree obtained for *k* in [15, 27] is topologically equivalent to the one obtained from our approach depicted in Fig. 5a, but the UWOPS034614 strain is clustered with the *S. paradoxus* clade rather than *S. cerevisiae* clade. Instead, for $k>40$, either the strain UWOPS919171 is misplaced or the stains Y12 and YPS128 are not grouped together, differently from the benchmark tree.

*Time and memory* Given the necessary data structures for this dataset, the former version phyBWT runs in approximately 12 min with a memory peak of 81 GB by loading the whole data structures in the main memory. The new version phyBWT2 uses only 6.5 MB of internal memory and fulfills the task in 3:12 min reducing the number of performed iterations from 6 to 1 (using the same parameter setting $k_m=22$ and $\tau =0.6$ for both versions). On the same datasets, using default parameters, SANS runs in 30:29 min by using 3.7 GB of internal memory. However, a direct time and memory comparison between phyBWT2 and SANS is not completely fair, as they take different inputs: if we do not assume availability of the data structures, computing them for this dataset takes over 3 hours, so SANS would be faster (see Table 2 for details).

### Experiments on *Drosophila*

Drosophila data are downloaded from the FlyBase database.^6^ This dataset includes assemblies from 12 species of the genus *Drosophila*: *D. melanogaster* (mel), *D. ananassae* (ana), *D. erecta* (ere), *D. grimshawi* (gri), *D. mojavensis* (moj), *D. persimilis* (per), *D. pseudoobscura* (pse), *D. sechellia* (sec), *D. simulans* (sim), *D. virilis* (vir), *D. willistoni* (wil), and *D. yakuba* (yak). Nine of these species fall within the Sophophora subgenus, which includes members of the melanogaster, obscura and willistoni groups.

The number of strings for each species varies: it ranges from 1, 870 for *D. melanogaster* to 17, 440 for *D. grimshawi*. The obtained dataset is a medium-sized input with a total number of symbols of more than 2, 161 Mbp. More details are reported in Additional file 1.

**Fig. 6:**
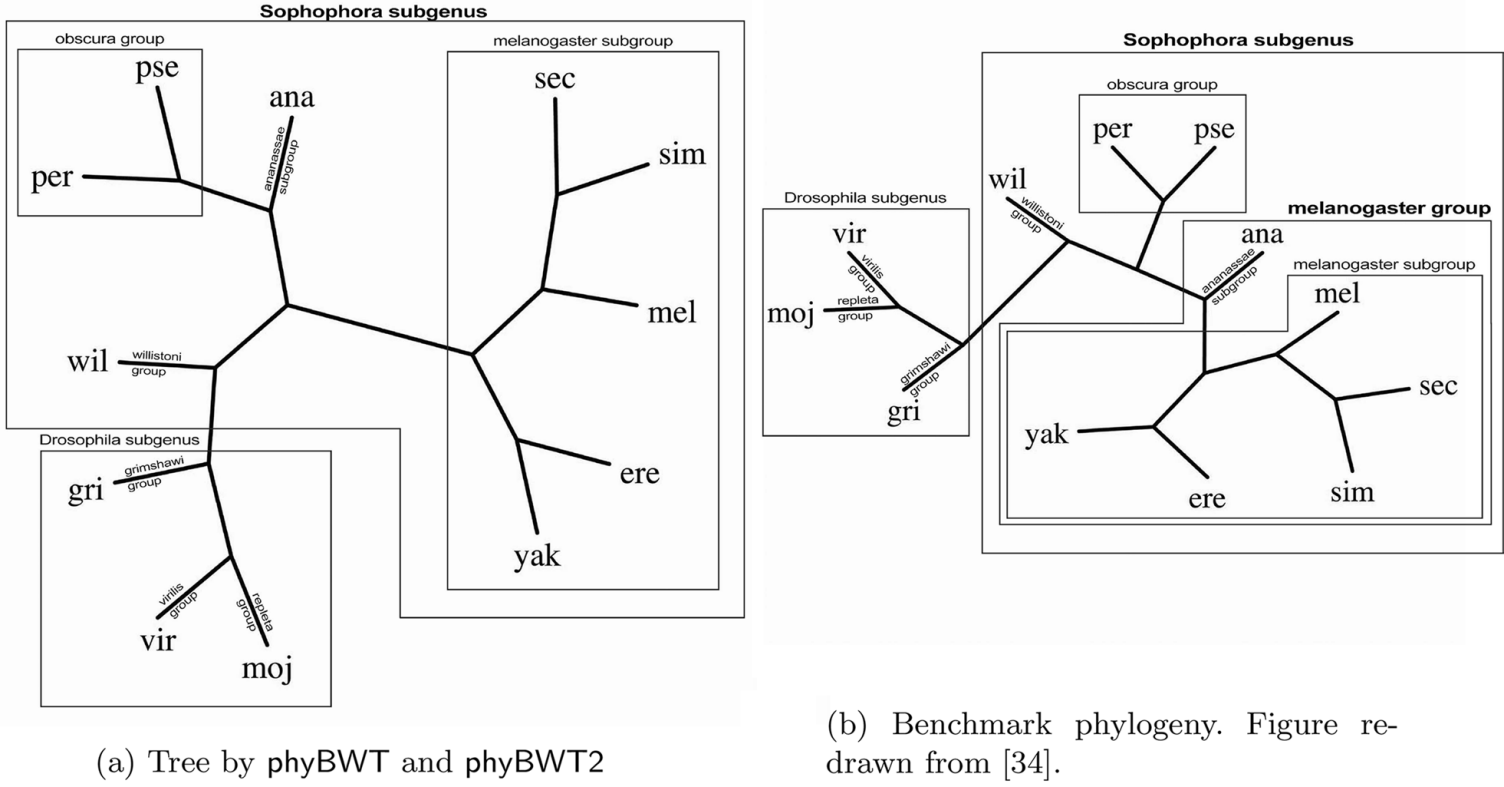
Drosophila phylogeny: **a** by our method; **b** benchmark redrawn from [34]

*Validation and results* As benchmark tree we used the accepted phylogeny [34] which we report in Fig. 6b. For this dataset, phyBWT2 produces the same tree as the one obtained in [11] by using phyBWT for any $k_m$ in [23, 45] and $\tau =0.5$ (Fig. 6a). The same parameter settings used for phyBWT also hold for phyBWT2. The *Sophophora* subgenus as well as the *Drosophila* subgenus are correctly detected, and inside the *Sophophora* subgenus, the *melanogaster* subgroup is correctly isolated. The only difference with respect to the benchmark tree by [34] is the taxon *D. ananassae* that represents the *ananassae* subgroup. Such subgroup is part of the *melanogaster* group together with *D. melanogaster*, *D. sechellia*, *D. simulans*, *D. erecta* and *D. yakuba*. However, our method places *D. ananassae* closer to the *obscura* group rather than the *melanogaster* subgroup. SANS was run with default values as described in [24], and the reconstructed tree obtained by option -f strict is topologically equivalent to the benchmark reference tree.

*Time and memory* phyBWT2 improves upon phyBWT by reducing the memory usage from 24 GB to 9 MB, and by reducing the number of iterations from 4 to 2 for $k_m=23$ and $\tau =0.5$. Nevertheless, the running time of phyBWT2 is around 9 min, more than phyBWT which ends in less than 2 min; this is mainly due to the fact that input data structures are kept on the disk. By using default parameters, SANS uses an amount of internal memory similar to phyBWT (28.7 GB) and ends in around 21 min.

### Experiments on 42 yeasts

In order to test the accuracy of phyBWT2 in discerning closely related populations (with sequence divergence varying between 0.5% and 1%), we selected 42 representative *S. cerevisiae* strains and produced their phylogenetic tree.

This dataset comprises 42 Illumina paired-end sequencing experiments obtained from the study in [52]. More details about the public repositories to download them are provided in Additional file 1. For each sequencing experiment, we extracted paired-end reads yielding an average coverage of $30\times$ per strain and a total FASTA file size of 38 GB.

**Fig. 7:**
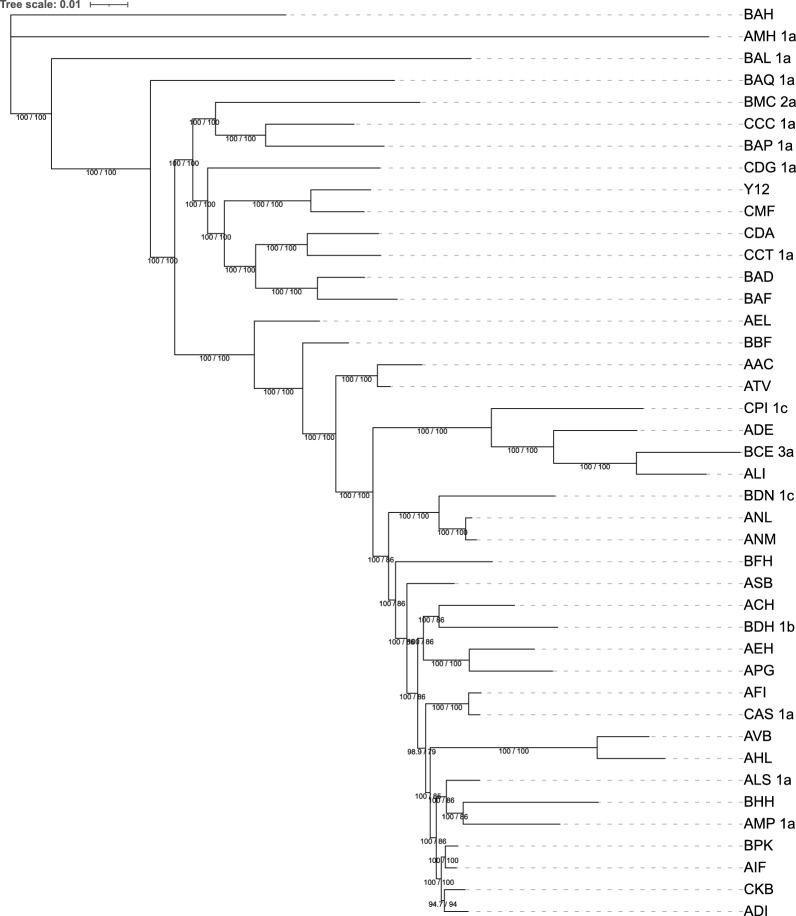
Bootstrapping phylogenetic tree on the 42 yeasts sequences

**Fig. 8:**
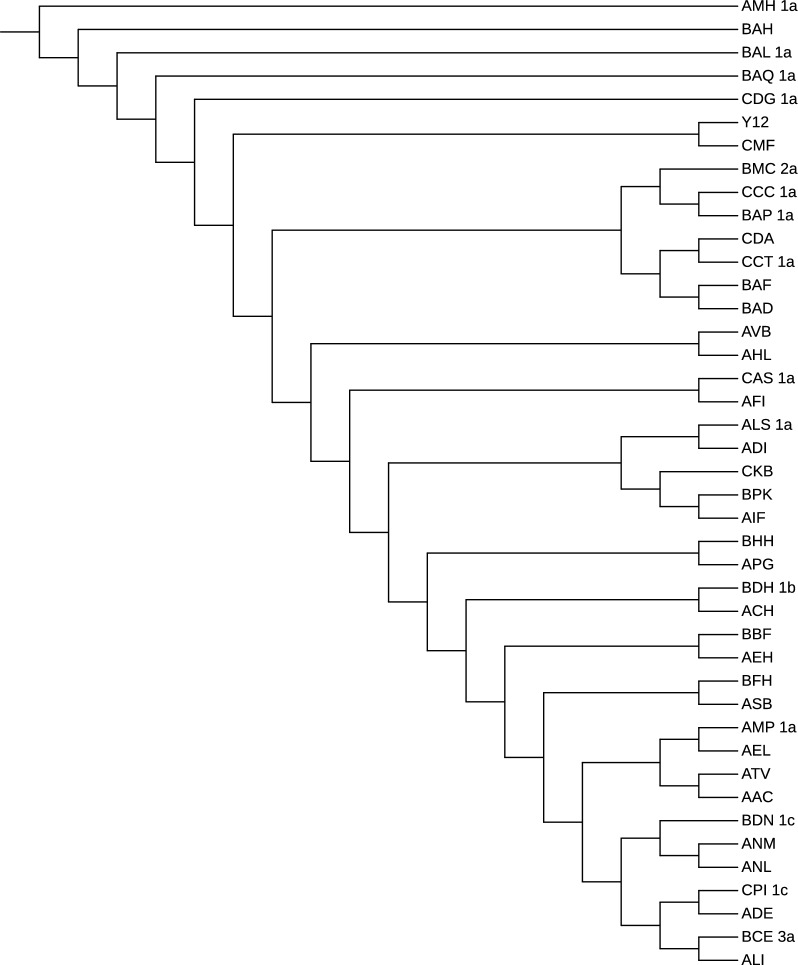
The phylogenetic tree on the 42 yeasts sequences by phyBWT

*Validation and results* As a benchmark we used IQ-TREE [53] with model selection [54] and ultrafast bootstrapping [55] (Fig. 7) and compared its results with those obtained from phyBWT2 and SANS. As shown in Fig. 8, phyBWT2 captured several features of the phylogeny produced with IQ-TREE.

Overall, the structure of the tree generated by phyBWT2 for sufficiently large $k_m$ and $\tau$ ($k_m=25$ and $\tau =0.6$) is very similar to the structure of the benchmark tree. All the main non-admixed clades [51], namely the “Wine/European” strains (AIF, BPK, ALS_1a, ADI, CKB, AFI, CAS_1a) and the Asian ones (BAQ_1a, BAH, BAL_1a, AMH_1a, BAP_1a), are correctly identified by phyBWT2. Remarkably, also the other two main classes of strains, namely the “ale beer” strains (AAC, ATV) and the “African palm wine” clade (BAD, BAF), are correctly clustered close to the Wine/European and the Asian clades respectively.

Finally, all the other strains related to the Wine/European clade are correctly clustered. Moreover, comparing the results of phyBWT2 with the literature [52] we observe that only phyBWT2 is able to correctly detect the outgroup strain (AMH_1a).

**Fig. 9:**
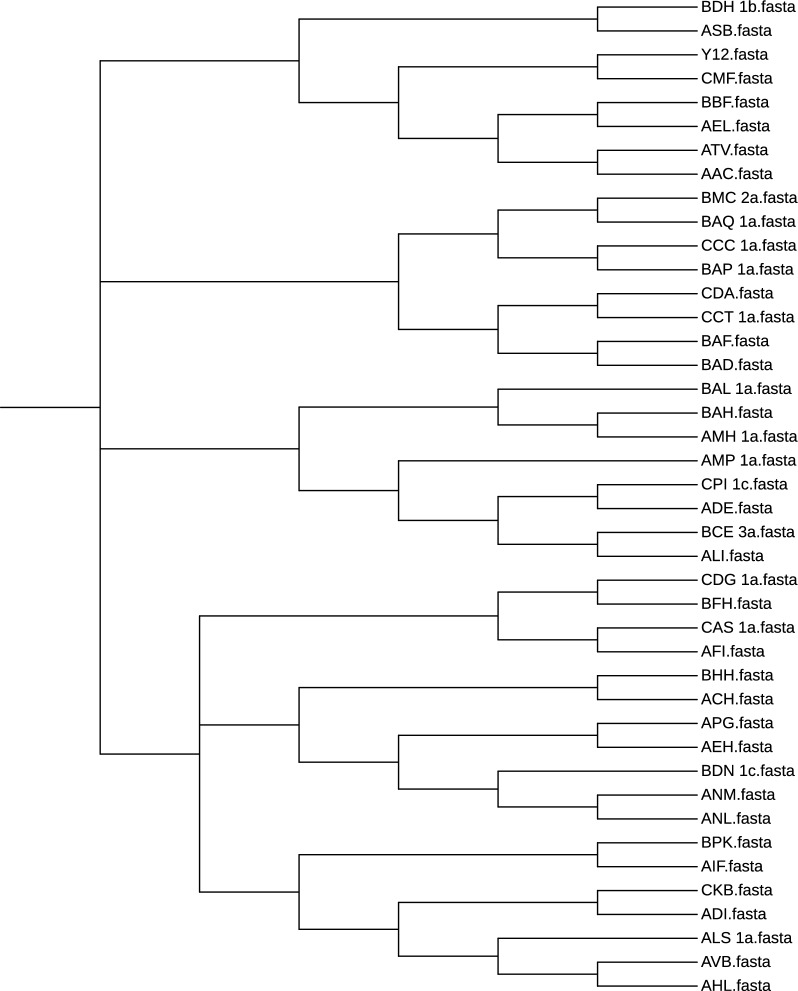
The phylogenetic tree on the 42 yeasts sequences by SANS with default parameters

On the contrary, the tree generated by SANS with default parameters (Fig. 9) fails to grasp both the general structure of the benchmark tree and the fine structure of the different clades. Remarkably, we observe strains related to the Wine/European group, such as the cider strain AMP_1a, clustered very close to Asian strains and also the other way round, e.g. the Asian strain CDG_1a that is clustered close to Wine/European strains.

**Fig. 10:**
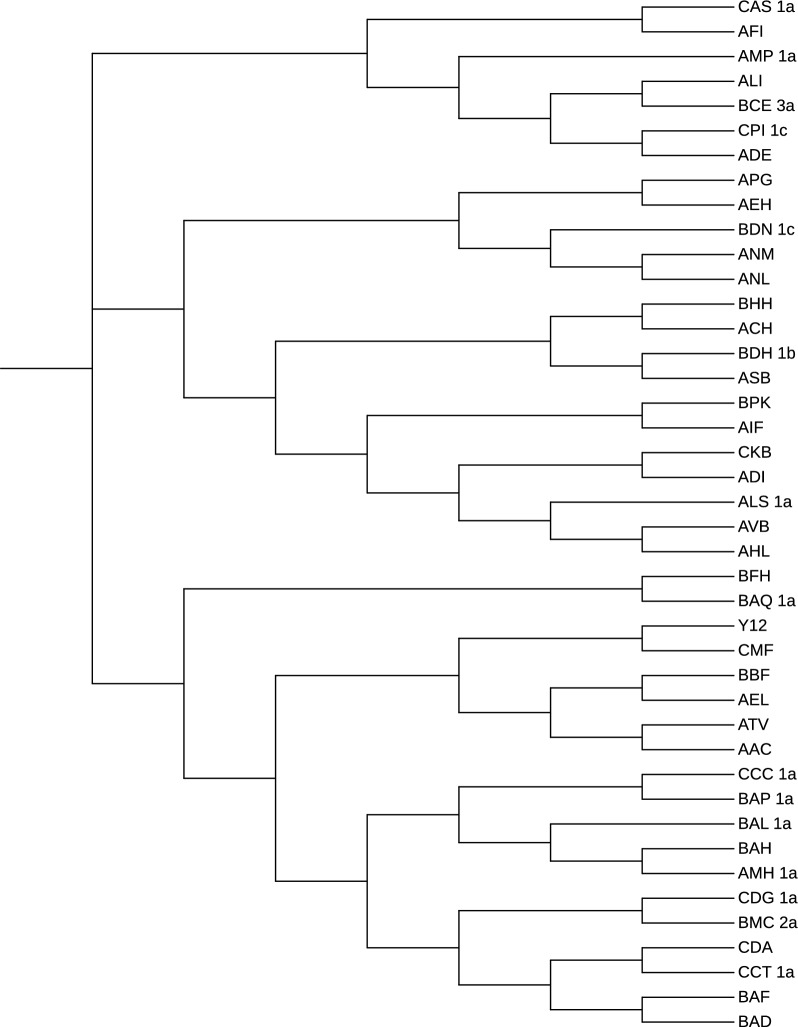
The phylogenetic tree on the 42 yeasts sequences by SANS with option -k=25

We also ran SANS using a smaller value for the *k*-mer length (option -k 25). As reported in Fig. 10 the general structure of the tree improved with respect to the default parameter, since the clustering of the non-admixed strains (both the Wine/European and the Asian) is correctly determined. Also the fine structure improved with the African palm wine strains (BAD and BAF) correctly clustered with the Asian clade. On the other hand, the ale beer strains (AAC and ATV) are still incorrectly placed. The same holds for the outgroup (AMH_1a) that is not identified.

*Time and memory* Given the necessary data structures for this dataset, phyBWT2 reconstructs the proposed phylogeny in approximately 1 h and half with a memory peak of 1.8 GB by using $k_m=25$ and $\tau =0.6$. phyBWT2 largely improves upon phyBWT not only but showing a better phylogeny reconstruction (by using similar parameter settings), but also in its performance. The former version runs in 5 h by using a large amount of memory (more than 175 GB) and performs 33 iterations against the only 5 carried out by phyBWT2. On the same dataset, SANS needs 13.5 GB of internal memory and more than 32 h to reconstruct the phylogenetic tree for $k=32$, and even more time (around more than 45 h) for $k=25$. Although a direct time and memory comparison between phyBWT2 and SANS is not fair, since phyBWT2 requires data structures whose computation does not depends on the choice of *k*, in this case even including the computation of the data structures phyBWT2 is more efficient than SANS: for this dataset, we can compute the eBWT and CDA array using the semi-external memory approach proposed by BCR [15] taking about 7 h and 4.3 GB of internal memory, and deduce the LCP array from the eBWT using the tool bwt2lcp [46] taking around 1 h and 20 min and 45 GB of memory (see Table 2).

### Experiments on HIV

Clade classification is an important task also in the field of virology, as each clade (also termed subtype) corresponds to a cluster of genetic similarity. Thus, we studied the phylogeny of the Human immunodeficiency virus (HIV).

There are two main types of HIV, and among them, HIV-1 is the most virulent and predominant. This dataset is obtained by selecting 43 HIV-1 complete genomes used in the literature [37]. In particular, it comprises thirty-five sequences from the major group (Group M) divide into subtypes A, B, C, D, F, G, H, J, K, seven sequences from the minor Groups N and O, and one CPZ sequence as an outgroup. Accession number, subtype, length (bp), and area of the HIV-1 sequences are reported in Table S1. These reference sequences have been carefully selected in [56] according to several criteria, and can be downloaded from the Los Alamos National Laboratory HIV Sequence Database.^7^

*Validation and results* For this experiment, we use as benchmark the phylogeny depicted in [37, Fig. 2], which is the Neighbor-Joining phylogenetic tree on the 43 reference sequences where the CPZ sequence (CIV strain AF447763) is used as an outgroup. We run phyBWT2 and SANS on this dataset by using different parameter settings. We compared the reconstructed phylogenetic trees by using the functions ClusteringInfoDistance() and SharedPhylogeneticInfo() provided in the R package TreeDist [57], which implements a suite of metrics to quantify the topological distance between pairs of unweighted phylogenetic trees.

**Fig. 11:**
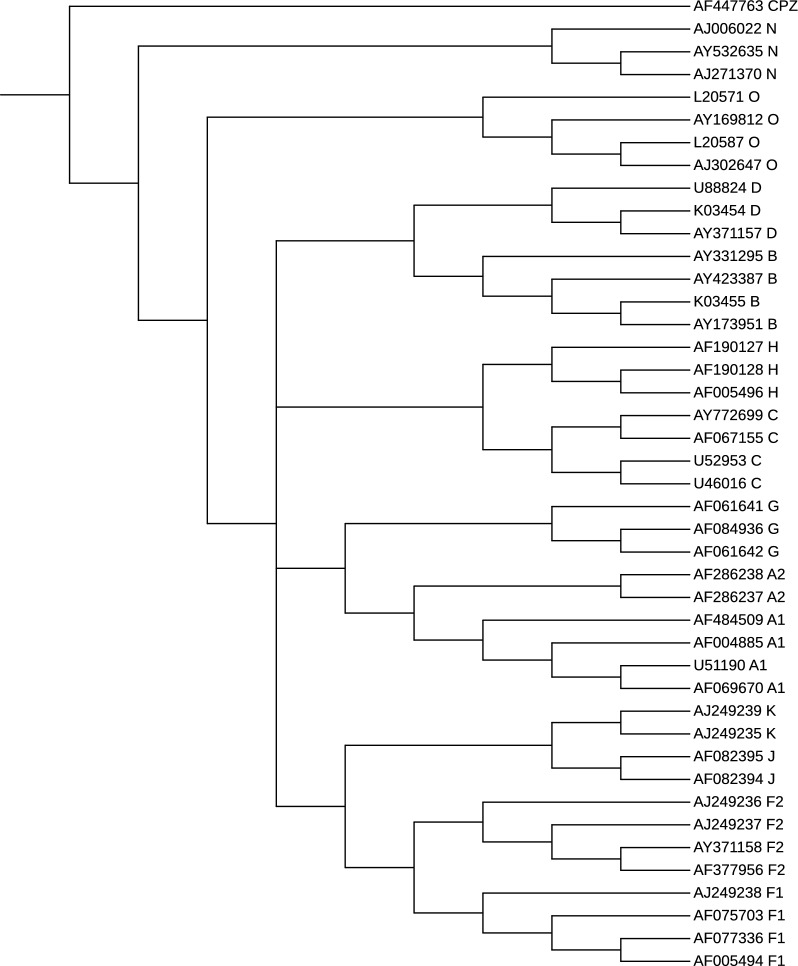
The phylogenetic tree on the 42 HiV-1 sequences by phyBWT2. Re-root the tree in CIV strain AF447763, as it is set outgroup in the reference tree in [37]

**Fig. 12:**
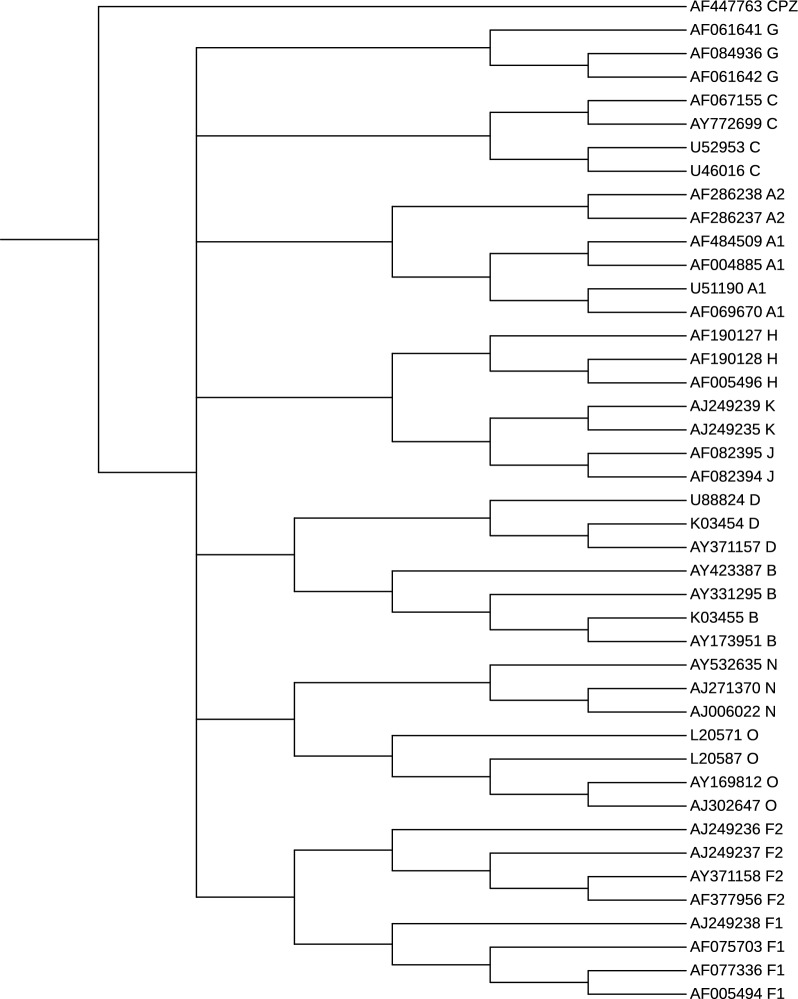
The phylogenetic tree on the 42 HIV-1 sequences by SANS. Re-root the tree in CIV strain AF447763, as it is set outgroup in the reference tree in [37]

Figures 11 and 12 depict the trees that obtained the best scores according to the above measures based on the amount of phylogenetic or clustering information that two trees hold in common. More in details, we set $k_m=16$ and $\tau =0.6$ in phyBWT2 and $k=16$ in SANS. For both tools, subtypes are distinctly grouped together in different branches. The phylogeny produced by phyBWT2 is consistent with the one in [37]. The relationships among the subtypes are well demonstrated, for instance subtypes B and D (resp. C and H) are closer to each other than to the others, and subtype F (resp. A) contains two distinguishable sub-subtypes F1 and F2 (resp. A1 and A2) that are close related to subtypes K and J (resp. G).

*Time and memory* Given the necessary data structures for this dataset, both phyBWT and phyBWT2 reconstruct the proposed phylogeny very quickly (less than 1 second). However, phyBWT2 improves on phyBWT by showing a phylogeny reconstruction closer to the benchmark philogeny (by using similar parameter settings), and by reducing the number of iterations from 8 to 3.

### Experiments on Ebolavirus

For this experiment, we used the 20 published sequences from [38] selected in [39].

The Ebolavirus genus includes five viral species: Ebola virus (*Zaire ebolavirus*, EBOV), *Sudan virus* (SUDV), *Tai Forest virus* (TAFV), *Bundibugyo virus* (BDBV), and *Reston virus* (RESTV). Ebola viruses are single-stranded RNA whose genomes consist of about 19 kilobases. Details for each sequence in Additional file 1.

*Validation and results* For this experiment, we use as benchmark the phylogeny trees depicted in [39, Fig. 4].

**Fig. 13:**
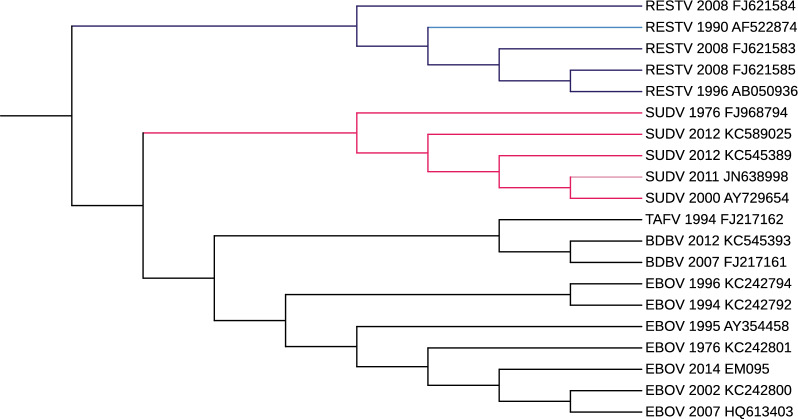
The phylogenetic tree on Ebolavirus dataset by phyBWT2

**Fig. 14:**
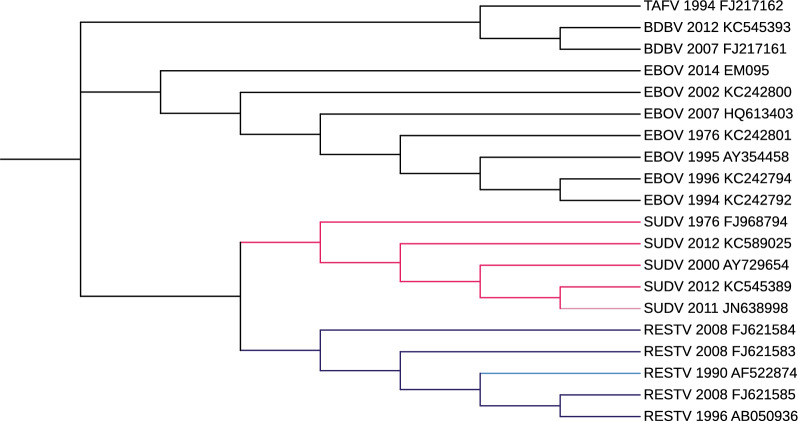
The phylogenetic tree on Ebolavirus dataset by SANS

Figures 13 and 14 depict the trees that obtained the best scores according to the measures based on the shared amount of phylogenetic or clustering information provided in the R package TreeDist [57] and described for the HIV-1 dataset. More in details, we set $k_m=16$ and $\tau =0.6$ in phyBWT2 and $k=21$ in SANS.

Both phyBWT2 and SANS exactly separated the five species. According to the four trees in [39, Fig. 4], the EBOV sequences are clustered into a monophyletic clade, and BDBV and TAFV viruses are positioned close and then clustered with the EBOV branch. These trees also show the phylogenetic uncertainty in the placement of the SUDV clade (red).

Our method (Fig. 13) places the SUDV clade as sister to the EBOV, TAFV and BDBV clade, in according with [39, Fig. 4E], whereas SANS (Fig. 14) places it as sister to RESTV clade in according with [39, Fig. 4A]. Differently from the study in [39, Fig. 4], the phylogeny reconstructed by SANS keeps the EBOV branch and the TAFV-BDBV clade separated (Fig. 14).

*Time and memory* Also in this second dataset of viral genomes, both phyBWT and phyBWT2 reconstruct the proposed phylogeny very quickly (less than 1 second). The phylogeny reconstructed by phyBWT is similar to the one produced by phyBWT2, but the number of iterations performed by phyBWT2 is much smaller (from 13 to 2).

### Experiments on *E. coli*—Shigella

For this experiment, we used a real-world dataset collected in the study [41] to assess the accuracy of the alignment-free methods in phylogenetic reconstruction of sequences that underwent horizontal gene trasfer events and genome rearrangements. It comprises 27 genomes of *E. coli* and *Shigella* whose reference supertree [40] was generated based on thousands of single-copy protein trees. Details for each sequence in Additional file 1.

*Validation and results* As benchmark we use the phylogeny tree depicted in [41, Additional file 2: Figure S8] where *E. coli* reference groups and *Shigella* (S) are indicated. Indeed, the 27 taxa are attributed to six distinct groups (the *E. coli* reference, or ECOR, strains) [40].

**Fig. 15:**
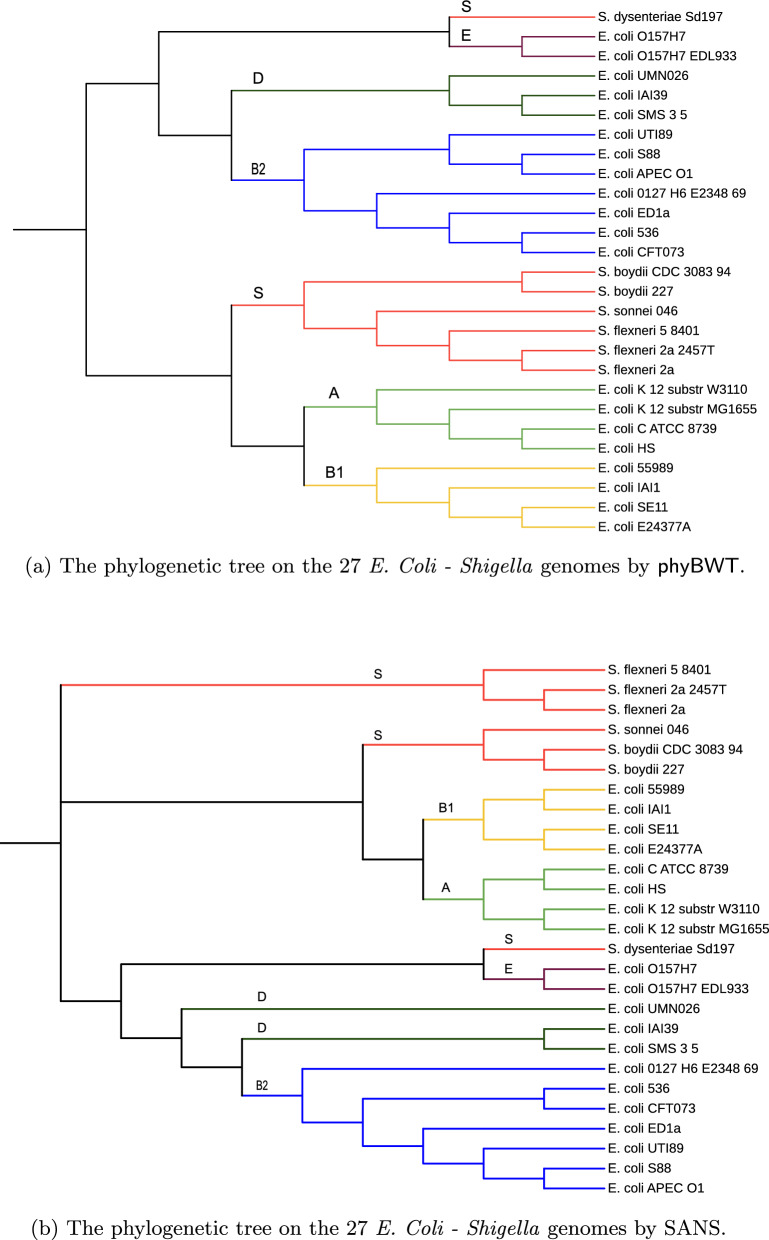
The phylogenetic tree on the 27 *E. coli—Shigella* genomes by phyBWT (**a**) and by SANS (**b**)

Figures 15a and b depict the trees we selected among them obtaining the higher scores according to the clustering information measure described above and computed by using the R package TreeDist [57]. More in details, we choose $k=16$ for SANS, and $k_m=16$ and $\tau =0.5$ for phyBWT2 (parameter setting similar to other datasets).

We observe that phyBWT2 clusters in clades each ECOR group (i.e. groups A, B1, B2, D, E and S), apart from *S. dysenteriae* that is placed externally to Group S, more precisely as sister to Group E.

However, we note that also in the reference tree *S. dysenteriae* is placed externally, as sister to the pathogenic *E. coli O157:H7* isolates (Group E). Also the tree reconstructed by SANS shows such a relationship between *S. dysenteriae* and Group E.

Differently from the reference tree, both phyBWT2 and SANS placed groups A and B1 as sister groups.

*Time and memory* The time usage of phyBWT 2 and SANS for this dataset is comparable (including the time for the preprocessing step completed by the internal memory approach gsufsort [45]), which is also comparable to the time spent by the older version phyBWT. By keeping the data structures on disk, phyBWT2 improves the memory usage of phyBWT. Finally, also for this datasets the number of iterations performed by phyBWT2 improves on those performed by the previous version.

## Conclusions and further work

In this paper, we proposed phyBWT2 an alignment-, assembly- and reference-free method to build the phylogeny inference of a set of taxa. The phyBWT2 method is a new version of phyBWT  [11] that includes improvements on the phylogenetic reconstruction strategy, as well as on the performance in both running time and memory usage. In fact, phyBWT2 is shown to reduce the number of iterations performed, and by keeping the data structures on disk it extremely reduces the main memory usage. To this extent, the implementation of phyBWT2 reads portions of $\textsf {lcp} (\mathcal {S})$, as well as of $\textsf {ebwt} (\mathcal {S})$ and $\textsf {cda} (\mathcal {S})$, and without the need of loading them in main memory, it performs the cluster detection by reducing on-the-fly the input data structures to any $\mathcal {R}\subset \mathcal {S}$ (i.e. by deducing $\textsf {lcp} (\mathcal {R})$, $\textsf {ebwt} (\mathcal {R})$ and $\textsf {cda} (\mathcal {R})$) from $\textsf {lcp} (\mathcal {S})$, $\textsf {ebwt} (\mathcal {S})$ and $\textsf {cda} (\mathcal {S})$.

To the best of our knowledge, phyBWT and phyBWT2 are the first methods that apply the properties of the Extended Burrows-Wheeler Transform (eBWT) to the idea of phylogenetic reconstruction. Both approaches are based on the eBWT positional cluster framework introduced in [19], which allowed us to consider longest shared substrings of varying length, unlike *k*-mer-based approaches such as SANS.

Differently from phyBWT, phyBWT2 combines the inner algorithm based on the eBWT positional clustering to a refinement procedure that reconstructs a phylogeny step-by-step by considering multiple partitions at each step, instead of just one partition as done by phyBWT.

We tested our method on several sequencing datasets, with short reads and de novo assembled sequences. The experimental results show that our algorithm produces trees comparable to the benchmark phylogeny and to the recently introduced tool SANS.

Our current implementation requires a preprocessing phase in order to compute the input data structures ($\textsf {ebwt}$, $\textsf {lcp}$ and $\textsf {cda}$), which are at the heart of several other text and string algorithms. Thus, evaluating the best tool or the combination of tools for the pre-processing phase is out of the scope of this work. More efficient tools for computing them can appear in the literature improving both the time and the memory requirements.

Moreover, the input data structures we used are independent of the parameter settings, so they can be computed only once and re-used for different runs of phyBWT2. Indeed, by using different types of data (e.g. genomes rather than short reads) phyBWT2 parameters may need to be fine-tuned, and there is no need of rebuilding from scratch the input data structures when changing phyBWT2 parameters. The same remarkable feature does not hold for *k*-mer-based approach, such as for instance SANS.

Phylogenetic analysis is a common practice in HIV studies [56, 58]. Experimentally we show phyBWT2 is able to distinctly group together the HIV-1 subtypes and to grasp the relationships among the subtypes. Virus subtypes can be clinically significant owing to their associations with variation in pathogenesis.

While the worst-case complexity of the method is competitive with existing methods, there are interesting directions for further optimization, such as using Colored Range Queries [59] to speed up identification of colors in the various clusters, or exploiting the bounded length of the reads to overcome the computational bottleneck of computing the eBWT and related data structures. A further improvement could include internally to phyBWT2 the bootstrapping of the reconstructed tree, for instance by ranging the value $k_m$ to vary eBWT positional clusters.

### Supplementary Information

**Additional file 1: **Dataset information. Descriptions and information to download the datasets used and analysed in the current study.

## Data Availability

The tool phyBWT2 is freely available for academic use at https://github.com/veronicaguerrini/phyBWT2. Information to download the datasets used and analysed in the current study is available as Additional file 1.

## Abbreviations

BWT: Burrows-Wheeler transform
eBWT: Extended Burrows-Wheeler transform
DA: Document array
LCP: Longest common prefix
SA: Suffix array
CDA: Color document array
SRA: Short reads archive
HIV: Human immunodeficiency virus
iTOL: Interactive tree of life

## Footnotes

1 https://github.com/veronicaguerrini/phyBWT.

2 Note that, in the implementations, one can use a single symbol as end-marker for all strings, but end-markers from different strings are then sorted on the basis of their index and the relative order of the strings in the collection they belong to.

3 In practice, we can stop at *X* instead of the root to consider fewer nodes, but the worst-case complexity is the same.

4 https://gitlab.ub.uni-bielefeld.de/gi/sans, downloaded in January 2023.

5 Except the trees in Figs. 4, 5, and 6 already in the conference paper [11].

6 http://flybase.org/

7 http://www.hiv.lanl.gov/

## Code availability

The source code is freely available for academic use at: https://github.com/veronicaguerrini/phyBWT2.
